# Dual-Stimuli-Sensitive Smart Hydrogels Containing Magnetic Nanoparticles as Antitumor Local Drug Delivery Systems—Synthesis and Characterization

**DOI:** 10.3390/ijms24086906

**Published:** 2023-04-07

**Authors:** Adam Kasiński, Agata Świerczek, Monika Zielińska-Pisklak, Sebastian Kowalczyk, Andrzej Plichta, Anna Zgadzaj, Ewa Oledzka, Marcin Sobczak

**Affiliations:** 1 Department of Pharmaceutical Chemistry and Biomaterials, Faculty of Pharmacy, Medical University of Warsaw, Banacha 1 Str., 02-097 Warsaw, Poland; 2 Faculty of Chemistry, Warsaw University of Technology, 3 Noakowskiego Str., 00-664 Warsaw, Poland; 3 Department of Toxicology and Food Science, Faculty of Pharmacy, Medical University of Warsaw, 1 Banacha Str., 02-097 Warsaw, Poland; 4 Military Institute of Hygiene and Epidemiology, 4 Kozielska Str., 01-163 Warsaw, Poland

**Keywords:** dual-stimuli-responsive hydrogels, magnetic hyperthermia, biodegradable polymers, antitumor drugs, paclitaxel, drug delivery systems, controlled release

## Abstract

The aim of this study was to develop an innovative, dual-stimuli-responsive smart hydrogel local drug delivery system (LDDS), potentially useful as an injectable simultaneous chemotherapy and magnetic hyperthermia (MHT) antitumor treatment device. The hydrogels were based on a biocompatible and biodegradable poly(*ε*-caprolactone-*co*-*rac*-lactide)-*b*-poly(ethylene glycol)-*b*-poly(*ε*-caprolactone-*co*-*rac*-lactide) (PCLA-PEG-PCLA, PCLA) triblock copolymer, synthesized via ring-opening polymerization (ROP) in the presence of a zirconium(IV) acetylacetonate (Zr(acac)_4_) catalyst. The PCLA copolymers were successfully synthesized and characterized using NMR and GPC techniques. Furthermore, the gel-forming and rheological properties of the resulting hydrogels were thoroughly investigated, and the optimal synthesis conditions were determined. The coprecipitation method was applied to create magnetic iron oxide nanoparticles (MIONs) with a low diameter and a narrow size distribution. The magnetic properties of the MIONs were close to superparamagnetic upon TEM, DLS, and VSM analysis. The particle suspension placed in an alternating magnetic field (AMF) of the appropriate parameters showed a rapid increase in temperature to the values desired for hyperthermia. The MIONs/hydrogel matrices were evaluated for paclitaxel (PTX) release in vitro. The release was prolonged and well controlled, displaying close to zero-order kinetics; the drug release mechanism was found to be anomalous. Furthermore, it was found that the simulated hyperthermia conditions had no effect on the release kinetics. As a result, the synthesized smart hydrogels were discovered to be a promising antitumor LDDS, allowing simultaneous chemotherapy and hyperthermia treatment.

## 1. Introduction

Tumor diseases are among modern medicine’s most difficult challenges. According to statistics, more than 19.3 million cancer cases were recently diagnosed, resulting in 10 million deaths worldwide in 2020 [1]. Despite tremendous progress in biomedical sciences in recent decades, the future prospects for tumor mortality are not hopeful. Furthermore, the average survival rate for most cancers is currently 5–7 years, and the chances of complete recovery are negligible. As a result, it is concluded that the efficacy of currently available antitumor treatments is insufficient. Among the possible solutions proposed to address the problem is the use of innovative nanoscale drug delivery systems (DDSs) and theranostics [1].

Among the most promising types of DDSs are hydrogels—3D, crosslinked polymeric structures capable of absorbing large amounts of water or biological fluids while retaining integrity [2]. The primary benefits of using hydrogels as drug carriers include ease of manufacturing and administration, the ability to deliver a variety of drugs, and nearly 100% drug entrapment [3]. Stimuli-sensitive hydrogels, also known as smart hydrogels, can change their properties in response to external or internal stimuli such as temperature, pH, ionic strength, electromagnetic radiation, magnetic field, or biological factors (antibodies or enzymes) [2,4,5]. As a result, they are being thoroughly examined as potential DDSs capable of releasing drugs in a prolonged and controlled manner. Furthermore, some of these biomaterials allow remote control of drug release; the drug can be released on demand [2].

Thermosensitive hydrogels are being investigated extensively as injectable local DDSs (LDDSs). The hydrogel forms a free-flowing sol at room temperature, which can be easily injected with a standard needle. Above the specific temperature known as the lower critical solution temperature (LCST), the hydrogel undergoes phase transition and forms a stable gel, due to physical crosslinking. As a result, when the hydrogel’s LCST is set slightly below physiological temperature, the system has the potential to be used as an injectable implant for drug delivery [6,7].

This strategy is highly beneficial; the drug depot can be easily implanted without surgical intervention, it prevents infections and scarring at the site of administration, and it increases patient comfort and compliance. If the polymer used to form the hydrogel is biodegradable or bioresorbable, the LDDSs do not require surgical resection after drug release [2,4,8,9,10]. Following intratumoral injection, the depot delivers the drug directly to the site of administration, limiting systemic distribution, reducing side effects, and enhancing therapy effectiveness.

In the field of thermosensitive smart hydrogels, polyesters and poly(ether-ester)s based on poly(*ε*-caprolactone) (PCL), polylactide (PLA) and polyglycolide (PGA) have gained much attention lately. These copolymers can form hydrogels with desired gelation behavior and physicochemical properties. Most importantly, they are biocompatible and biodegradable [2,11,12], which are highly desirable for biomedical applications. Furthermore, they are easily synthesized in a one-step ring-opening polymerization (ROP) process using cyclic ester monomers and a diol as an initiator [13,14]. These copolymers have an amphiphilic structure comprising hydrophobic (A) blocks (polyester) and hydrophilic (B) blocks (diol). Thus, in aqueous media, the ABA and BAB triblock copolymers can self-assembly into micelles with temperature-dependent gel-forming properties [6,7]. Furthermore, amphiphilic macromolecules can act as a solubilizer for hydrophobic and insoluble drugs, significantly increasing their water solubility [15]. To date, a considerable amount of thermosensitive hydrogels based on PCL/poly(ethylene oxide) (PEG) [16,17,18,19,20], PLA/PEG [21,22], PCL/PLA/PEG [3,23,24], and PCL/PGA/PEG [25,26] block copolymers have been described in the literature as potential DDSs with attractive properties. 

Hyperthermia is a novel antitumor treatment that is gaining in popularity. This method involves heating a part of or the entire body to 41–45 °C for a predetermined period of time [27]. Hyperthermia is classified as local hyperthermia, which affects a single tissue, regional hyperthermia, which affects an organ, and whole-body hyperthermia [28]. According to antitumor therapy, local hyperthermia appears to be preferable. Furthermore, it is usually combined with chemotherapy or radiotherapy to increase treatment efficiency in a synergistic manner; hyperthermia increases tumor sensitivity to cytostatics and reduces drug resistance [28,29].

Hyperthermia can alter cellular physiology in a variety of ways. Heat shock and apoptosis are the two main pathways by which the changes occur. Heat shock activates heat shock proteins, resulting in protein unfolding and loss of functionality. Furthermore, heat shock proteins cause an immune response. The apoptosis pathway is activated by specific cytokines and caspases, resulting in programmed cell death that does not cause inflammation. Specific markers are expressed on the tumor cellular membrane as the temperature increases, tends to result in immunological recognition. Furthermore, tumor antigens are secreted as a result of exosome secretion, activating the immune system [28]. An increase in temperature between 41 °C and 44 °C leads cancer cells to the apoptotic pathway in a highly selective manner, leaving healthy cells unaffected [28,30]. Thereby, when combined with other therapies, hyperthermia was found to be a very promising method of antitumor treatment because it is well tolerated and increases the effectiveness of the therapy [28]. The most dependable mode of hyperthermia application, according to the literature, is multiple cycles of heating the tumor site for approximately 1 h. Because tumor cells develop temporal thermoresistance in response to increased temperature, the time interval between hyperthermia episodes should be at least 48–72 h [31,32]. 

Among other techniques for inducing local hyperthermia, alternating magnetic field (AMF)-mediated hyperthermia using magnetic nanoparticles appears to be the most promising and beneficial. The magnetic field has excellent tissue permeability and is minimally invasive; AMF strength can be easily modified and temperature elevation can be controlled remotely [28,33]. The nanoparticles can be injected intravenously, allowing for the homogenous distribution of magnetic material throughout the tumor. Moreover, the particles tend to retain in the administration side without significant leaking [33,34]. As a result, the heat generated during the hyperthermia procedure is precisely dissipated in the administration site, sparing the surrounding tissues and reducing side effects [33]. The most widespread class of nanoparticles used for magnetic hyperthermia therapy (MHT) are magnetic iron oxide nanoparticles (MIONs).

MIONs are a class of Fe(II) and Fe(III) particles in various forms, such as maghemite (g-Fe_2_O_3_) or magnetite (Fe_3_O_4_), that have magnetic properties with low coercivity (*H*_c_) and remanence (*B*_R_), due to their nanoscale size (5 nm–20 nm core diameter), resulting in single-domain and superparamagnetic behavior. They are not magnetized in the absence of an external magnetic field [35]. In the presence of AMF, MIONs can convert magnetic field energy into heat via Néel or Brown relaxation mechanisms, which are based on the reorganization of magnetic moments or the rotation of whole particles, respectively, due to changes in the external magnetic field [29]. 

Despite having negligible *H*_c_ and *B*_R_, MIONs tend to agglomerate in aqueous media, which is highly undesirable for biomedical applications. Thus, the particles are typically surface modified with various polymers to limit agglomeration, prevent Fe(II) to Fe(III) oxidation, and ensure biocompatibility [29,36,37].

MIONs have been extensively studied in recent years and used for a variety of potential biomedical applications, including regenerative medicine, drug and gene delivery, diagnostic and theranostic agents, and magnetic hyperthermia-inducing devices [36,38]. There is also literature evidence that MIONs can be used for antitumor therapy. Toraya-Brown et al. [39] administered starch-coated iron oxide nanoparticles into tumor-bearing mice. Following AMF application, significant tumor cell damage was observed due to hyperthermia, with no damage to vital organs. Hardiansyah and coworkers [40] obtained magnetoliposomes containing MIONs for controlled release of doxorubicin (DOX) and magnetic hyperthermia induction in the presence of AMF. The cytotoxicity against colorectal cancer cells was significantly increased, indicating a synergistic effect of chemotherapy combined with hyperthermia [36]. Notably, coated MIONs are biocompatible, according to in vivo animal experiments and clinical trials; they are also used as an MRI contrast agent. MION toxicity is dose-dependent, and it was observed at doses far in excess of the dose required to induce magnetic hyperthermia [35].

The concept of MIONs-mediated magnetic hyperthermia for antitumor therapy, as well as hydrogel DDSs releasing cytostatics, is well known. Nonetheless, the evidence for MIONs combined with smart hydrogel antitumor DDSs is extremely limited, particularly in biodegradable and poly(ether-ester)s-based matrices. Furthermore, smart hydrogel systems for antitumor drug delivery are not used in clinical practice. However, there have been reports that chemotherapy and hyperthermia can work together to significantly increase the efficacy of the therapy [2,41,42,43]. 

Because of the foregoing, the dual-stimuli-responsive smart hydrogel LDDSs—temperature and AMF sensitive, are presented in this paper as a potential carrier of the model antitumor drug paclitaxel (PTX). The system allows for simultaneous hyperthermia treatment and innovative chemotherapy with a controlled drug release profile. The injectable hydrogel was formed from biodegradable and biocompatible poly(ether-ester): poly(ε-caprolactone-*co-rac*-lactide)-*b*-poly(ethylene glycol)-*b*-poly(ε-caprolactone-*co-rac*-lactide) (PCLA-PEG-PCLA, PCLA), synthesized via ROP [23,44]. Due to its lower toxicity, zirconium(IV) acetylacetonate was used as a catalyst instead of the more commonly used stannous octoate [45,46]. The structure of the synthesized copolymers and their molecular weight were studied in detail using ^1^H and ^13^C NMR as well as gel permeation chromatography (GPC) techniques; the gel-forming properties of the hydrogels, as well as their cyto- and genotoxicity, were investigated. The synthesis procedure of the hydrogel with preferable properties was denoted based on the collected data. The coprecipitation method was used to obtain MIONs [47,48,49,50], which were thoroughly studied using transmission electron microscope (TEM) and dynamic light scattering (DLS) measurements; optimal synthesis and coating conditions were also defined. The magnetic properties of the nanoparticles were assessed using a vibrating sample magnetometer (VSM), as well as their ability to induce hyperthermia. Following that, in the final step of the research, the LDDS comprised of the thermosensitive hydrogel and MIONs was used as a PTX carrier; the drug release profiles in various conditions were measured, and the obtained data were fitted to mathematical models to consider the kinetics and mechanism of the drug release.

## 2. Results and Discussion

### 2.1. PCLA Synthesis and Optimization

**Figure 1:**
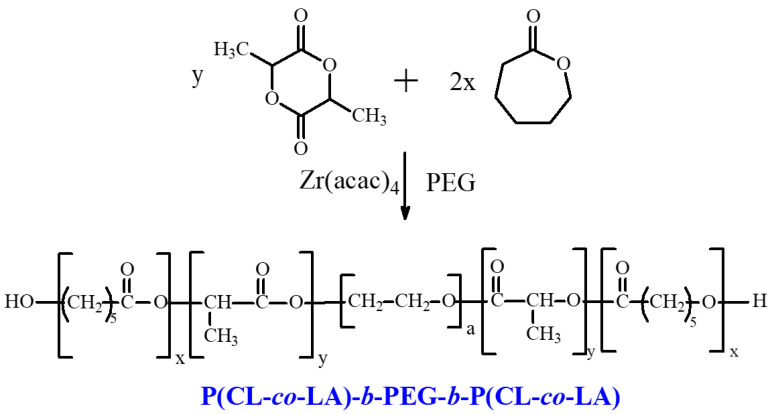
The PCLA synthesis via the ROP process.

**Figure 2:**
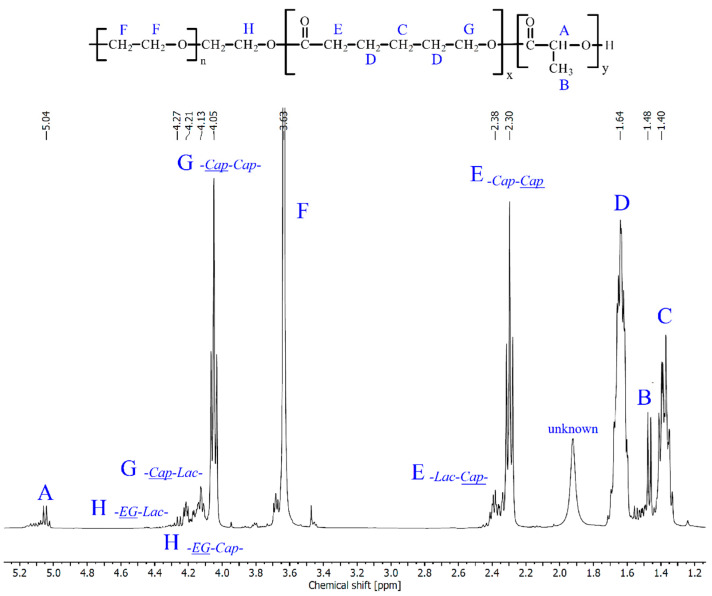
^1^H NMR spectrum of the PCLA copolymer.

**Figure 3:**
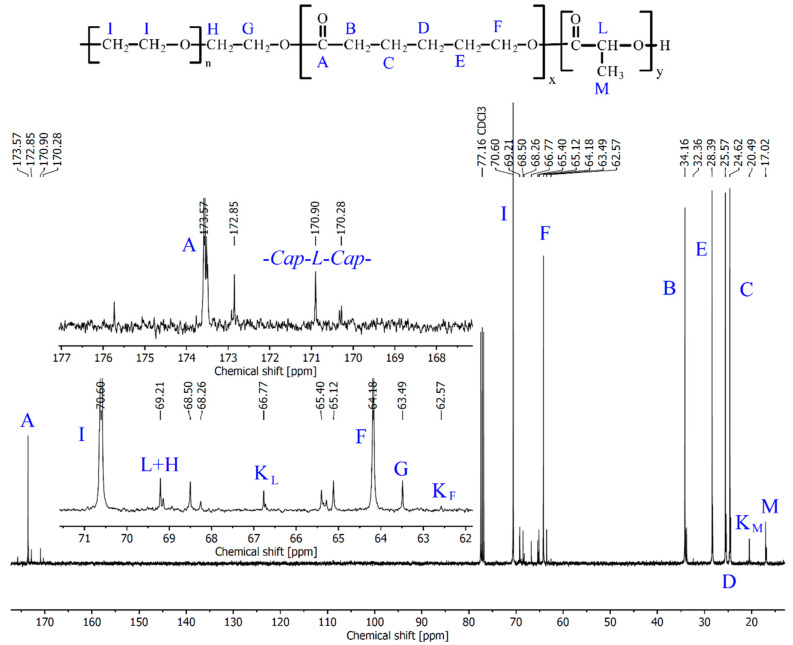
^13^C NMR spectrum of the PCLA copolymer. K_M_, K_F_ and K_L_ refer to the M, F and L atoms in the copolymer chain end groups, respectively.

In the first stage of the research, a series of polyester and poly(ether-ester) syntheses were performed to determine the optimal process conditions, which included temperature, reaction time, and the monomer/catalyst molar ratio. The PCLA copolymers were successfully synthesized using the ROP process from *ε*-caprolactone (CL), *rac*-lactide (LA), and PEG (Figure 1), as confirmed by ^1^H and ^13^C NMR analysis. The copolymer’s characteristic signals were clearly visible on the recorded spectra and were consistent with literature data [44,51]. The example spectra are shown in Figure 2 and Figure 3, and their interpretation is detailed in the Section 3.

The signal visible on the ^13^C NMR spectrum at the chemical shift of 170.90 ppm corresponds to the carbonyl carbon atom from lactyl (*L*) groups in -*Cap-L-Cap*- sequences. These sequences cannot be formed as a result of ROP of lactide, which normally results in lactydyl (*LL*) sequence formation. Their presence in the polymer chains is due to intermolecular transesterification processes [44]. 

Table 1 contains the data from the PCLA syntheses at the conditions optimization stage. The syntheses were carried out using 1.0 g of PEG 1500. The CL/PEG [m/m] and LA/PEG [m/m] ratios were constant and both equal to 1.1. 

In general, the conversion of LA was found to be significantly higher than the conversion of CL in all of the synthesized samples; the characteristic signals from LA monomer in the recorded ^1^H NMR spectra of post-reaction mixtures are almost invisible. As a result, we can assume that LA is more reactive in this type of synthesis, and the first mer connected to the PEG terminal hydroxyl group is typically LA. These findings are consistent with the literature [44].

The main parameters considered during the ROP optimization stage were the agreements between the number of CL or LA mers in the synthesized polymeric chains (calculated from ^1^H NMR spectra) and the feed ratio. These parameters would be perfect in an ideal situation. Furthermore, high CL conversion was discovered to be necessary for optimal polymerization conditions.

According to the data collected, CL conversion increased in tandem with reaction time (samples PCLA-1, PCLA-2, PCLA-3, PCLA-12 and PCLA-13). When the monomer/catalyst molar ratio was 1000:1, sufficient CL conversion (above 90%) was observed after 24 h and 6 h at 130 °C and 150 °C, respectively. After 24 h, the reaction at 110 °C achieves a CL conversion of 74%. It was also discovered that the increase in temperature increased CL conversion (samples PCLA-1 and PCLA-4, PCLA-2 and PCLA-5, PCLA-6 and PCLA-12) for all reaction times studied. However, the greatest temperature influence was observed for the shorter ones, namely 3 and 6 h. After 24 h, CL conversion reached 74%, 91%, and 94% at 110 °C, 130 °C, and 150 °C, respectively. Because the CL conversion is high over such a long period of time, the effect of temperature is less noticeable.

In general, increasing the monomer/catalyst molar ratio reduced CL conversion. When the ratio was reduced from 1000 to 500 (or 750) for the samples PCLA-7–PCLA-10 and PCLA-13 and PCLA-14, CL conversion increased from 91% to 98% and from 94% to 98% at 130 °C and 150 °C, respectively.

The critical parameters considered in the ROP reaction optimization stage were the theoretical and experimental CL and LA mer numbers. The agreements were approximately 120% for CL and 95% for LA for the samples with sufficient CL conversion (>90%). Furthermore, the synthesis demonstrated high reproducibility (samples PCLA-8 and PCLA-9, Table 1). According to the data collected, the parameter agreements are satisfactory and appear to be independent of the investigated reaction conditions when monomer conversion is high. The sample PCLA-12 was found to be an exception to the rule. The poor agreement for CL mers is thought to be due to the high temperature (150 °C) and short reaction time (6 h). These conditions resulted in a high rate of PCL formation, which was initiated by molecules of the water traces or CL itself. Transesterification processes connected the PCL chains to the PCLA chains, resulting in a higher amount of CL in the polymeric chains.

Based on the average length of -*Cap-Cap*- sequences (*LCap*), it is possible to conclude that the microstructure of hydrophobic blocks is random. The calculated *LCap* values for all of the synthesized samples were low (approximately 2 in most cases). As a result, no distinct blocks of PCL or PLA can be distinguished within the copolymer hydrophobic blocks, indicating the statistical microstructure of the chains. Furthermore, the random microstructure of the blocks is caused by the intermolecular transesterification processes that occur during synthesis. The random microstructure of the hydrophobic blocks is noteworthy because it reduces polymer crystallinity, which improves hydrogels stability and PCLA water solubility [52,53].

According to the data, the optimal PCLA synthesis conditions in the ROP reaction were 130 °C, 24 h and a monomer/catalyst ratio of 750:1. These conditions resulted in good process reproducibility, high monomer conversion, and satisfactory agreement between the theoretical and experimental average numbers of CL and LA mers in the PCLA copolymeric chain. As a result, subsequent syntheses were performed under the aforementioned, optimized conditions. 

The following step in the investigation was a series of syntheses to determine the optimal copolymer structure, which would provide the product with the appropriate gel-forming properties. The incorporation of LA into the PCL hydrophobic blocks of the PCL/PEG copolymer reduces PCL crystallinity, improving its water solubility and hydrogels stability. As a result, a series of PCLA copolymers were synthesized under optimized conditions, with varying CL/PEG and LA/PEG ratios (using 1.0 g of PEG 1500); the results are shown in Table 2.

The ratios of the LA and CL mers were planned in two ways. The hydrophobic chains in the first method were formed primarily from PCL, which was slightly modified with a small amount of LA (PCLA-15–PCLA-18, Table 2). The CL/LA ratios in the second method were close to 1; the amounts of CL and LA in the PCLA hydrophobic chain were comparable.

The PCLA copolymers were successfully synthesized, and the final product was either a white, amorphous solid (samples PCLA-15–PCLA-18) or a greasy, viscous liquid (PCLA-19–PCLA 25), depending on the LA/CL ratio. The agreement between the theoretical and experimental average numbers of CL and LA mers in the resulting polymers was close to 100% and comparable to the values from the synthesis conditions optimization stage (Table 1).

The LCST and the upper critical solution temperature (UCST) of the formed hydrogels are critical parameters in determining the optimal copolymer structure. The temperatures were measured using the tube-inverting method. As shown in Table 2, all of the synthesized PCLA copolymers dissolve in water, forming thermosensitive, smart hydrogels with a LCST in the 27–34 °C range. The most promising gel-forming properties were observed for samples PCLA-15 and PCLA-20, according to a desirable hydrogel LCST slightly below 37 °C and an UCST slightly above 41 °C, required for potential application as thermosensitive, injectable hydrogels used for hyperthermia therapy. As a consequence, the CL/PEG [m/m] and LA/PEG [m/m] ratios used for larger-scale copolymer synthesis and subsequent experiments were 1.8 and 0.3 (sample PCLA-15) or 1.1 and 1.2 (sample PCLA-20), respectively.

In a larger scale, the two optimal PCLA samples were synthesized using the described procedure (samples PCLA-A1 and PCLA-A2). The scale increase had no significant effect on the structure and properties of the obtained copolymers (Table 2).

The number average molecular weight (*M_n_*) and dispersity (*Đ*) values of the optimized copolymers synthesized at a larger scale were evaluated using GPC method. The results were then compared to microscale samples of PCLA-15 and PCLA-20. Table 3 displays the outcomes.

The data show that the observed *M_n_* values are slightly higher than the ^1^H NMR calculations (Table 2). However, the dispersity of the examined samples was narrow (approximately 1.3), demonstrating good reaction control and the efficacy of the copolymer purification process. Furthermore, the results obtained for microscale and larger-scale synthesis were highly comparable; the scale extension had no significant effect on the product’s properties.

### 2.2. Sol–Gel–Sol Transition of the PCLA Hydrogels 

**Figure 4:**
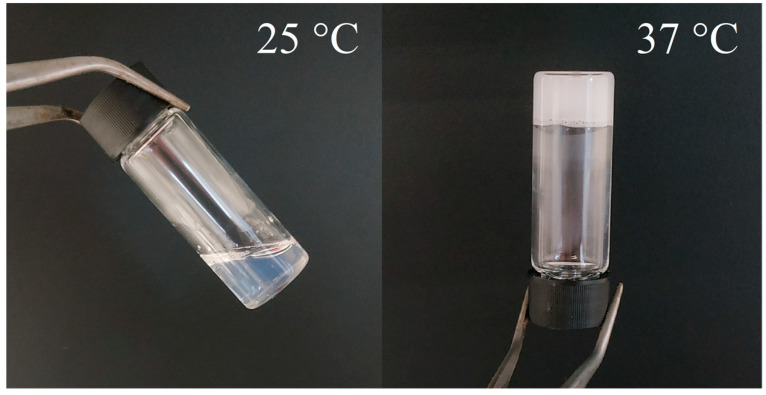
PCLA hydrogel (25 wt.%) behavior at 25 °C and 37 °C.

Table 2 shows that all of the synthesized copolymers dissolve in aqueous media and form transparent, free-flowing sols at room temperature. When the temperature remained at 25 °C or 4 °C, the hydrogel matrices remained stable and did not spontaneously form a gel. Furthermore, when the system temperature was raised to 37 °C, the product became white, opaque, and formed a semisolid gel (Figure 4).

The LCST and the UCST of the hydrogels were determined using the tube-inverting method. In general, increasing the copolymer *M_n_* increases the LCST and the UCST of the hydrogels, shifting the gel window towards higher temperatures (samples PCLA-24 and PCLA-25, Table 2). When the copolymer *M_n_* is higher, there are fewer copolymer molecules in an aqueous solution of a given percent concentration. As a result, the number of the formed micelles is reduced, resulting in an increase in the LCST value [16]. On the other hand, it is known that increasing the hydrophobic block in poly(ether-ester) triblock copolymers results in a decrease in the LCST value and a widening of the hydrogel’s gel window due to stronger hydrophobic interactions between the micelles [6,44]. When the number of hydrophobic sequences increases, the hydrophobic/hydrophilic balance of the macromolecule also increases. Thus, the stabilization effects from the hydrophilic moieties are reduced, and the sol–gel phase transition of the hydrogel is facilitated, resulting in a decrease in the LCST. Furthermore, the increase in the hydrophobic/hydrophilic balance decreases the aggregation number of the micelles. When the micelles are created by fewer molecules, more of them are formed (at given concentration of the copolymer), leading to a reduction in the LCST. Nevertheless, because the hydrophilic blocks of the synthesized PCLA were mostly derived from PEG 1500 in all samples, the increase in copolymer *M_n_* is inextricably associated with the increase in its hydrophobic blocks. As a result of the contradictory effects of these changes, the determination of the copolymer *M_n_* and the size of hydrophobic blocks influence on the gel window appears to be intractable.

Interestingly, increasing the LA/CL ratio leads to a decrease in the LCST (PCLA-15, PCLA 19, PCLA-20, PCLA-21, Table 2) when the *M_n_* of the copolymers is constant.

**Figure 5:**
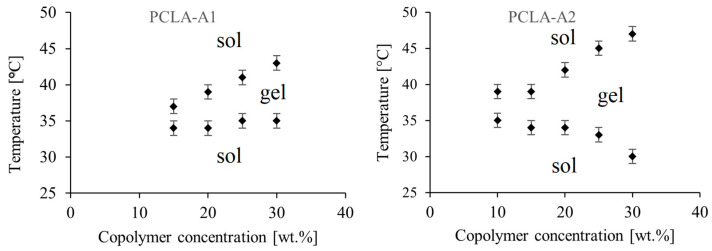
Phase transition diagrams of the PCLA hydrogels (PCLA-A1 and PCLA-A2).

The gel-forming properties of the PCLA hydrogels were thoroughly investigated for the larger-scale samples (PCLA-A1 and PCLA-A2, Table 2). For various copolymer concentrations (5 wt.%–30 wt.%), the LCST and the UCST, as well as the critical gelation concentration (CGC), were determined. Figure 5 depicts the results.

**Figure 6:**
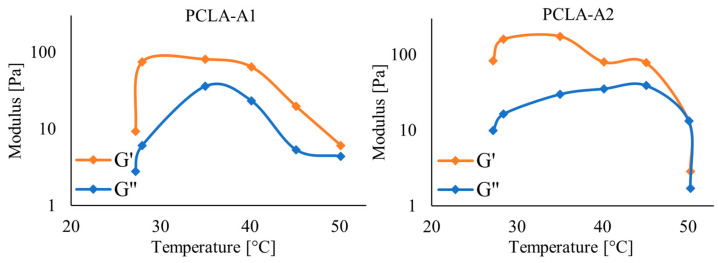
G’ and G” data of the PCLA-A1 and PCLA-A2 hydrogels as a function of temperature.

The behavior of the obtained hydrogel samples is similar to that of the corresponding microscale samples; the samples were dissolved in water and formed transparent sols at room temperature at all concentrations tested. The gel window expanded as the copolymer concentration increased, indicating a decrease in the LCST and an increase in the UCST values. This phenomenon is typical in poly(ether-ester) hydrogels and is associated with a higher concentration of copolymer macromolecules in the hydrogel sample [6]. The effect was more noticeable for the PCLA-A2 hydrogel because of the significant difference in the copolymer structure; the PCLA-A2 sample contained a higher amount of CL in the hydrophobic block of the macromolecule (Table 2). Furthermore, the minimal concentration at which the sol–gel transition occurred in the PCLA-A2 hydrogel was lower; the observed CGC was 10% (compared to 15% for the sample PCLA-A1). The storage modulus (G’) and loss modulus (G”) values of the PCLA-A1 and PCLA-A2 hydrogel samples were measured at 27–50 °C using a rotational rheometer. Figure 6 displays the outcomes.

According to the plots, the G’ and G” values increased during the initial heating stage, with the increase being more evident for the PCLA-A1 hydrogel. This fact appears to be due to a higher LCST value in the PCLA-A1 sample. It is worth noting that the maximum values of the moduli appear to be sufficient and are observed at temperatures close to 37 °C, implying that the mechanical properties of the hydrogels are satisfactory. When the temperature increases above the UCST, the micelles decompose and copolymer precipitation occurs, resulting in a decrease in moduli values. The produced PCLA hydrogels’ sol–gel–sol transition behavior is considered to be ideal for biomedical applications as thermosensitive, injectable LDDSs.

### 2.3. Cyto- and Genotoxicity Assay

Following the appropriate guidelines [13,54], the obtained PCLA matrices were subjected to a neutral red uptake (NRU) assay to evaluate their cytotoxicity. At the highest concentration (1 mg/mL), the viability of the BALB/c 3T3 cells for the PCLA-A1 and PCLA-A2 copolymers was 97% and 107%, respectively. In comparison to untreated cells, none of the samples at any of the tested dilutions had cell viability less than 70%. As a result, it was determined that the PCLA matrices are not cytotoxic. Interestingly, the observed viability was even higher when compared to untreated cells. One possible explanation for this phenomenon is that the products of the copolymer hydrolysis act as nourishment for the test cells.

The *umu* test, on the other hand, proved the absence of genotoxicity for PCLA samples [13,55]. The tested copolymers, even at the highest concentration (1 mg/mL), had no toxic effects on *Salmonella typhimurium* TA1535, regardless of growth factor or metabolic activation. Furthermore, the induction ratio (IR) values for the tested samples were significantly lower than 1.5, indicating that the PCLA copolymers were not genotoxic. Tables S1 and S2 show the detailed results of the toxicity assays (Supplementary Materials).

### 2.4. MIONs Synthesis and Characterization

According to the literature, MIONs can be synthesized by coprecipitation via two different routes. In the first method, an alkaline solution of ammonia or sodium hydroxide is added to an aqueous magnetically or mechanically stirred mixture of Fe^2+^ and Fe^3+^ salts [56]. Alternatively, the iron salts solution can be mixed with the alkaline solution. Based on Smolkova et al. study [48], the latter method of synthesis yields better results; the obtained nanoparticles are almost spherical, the size and size distribution is lower, and the saturation magnetization is higher when compared to MIONs obtained by the former method. This phenomenon can be explained by the abrupt pH changes that occur during the synthesis when the alkaline solution is added to the iron salts mixture, as well as the more complicated mechanism of magnetite formation. As a result, in our study, the MIONs were synthesized by dissolving the iron salts mixture in alkaline solution.

In the first step, a series of MIONs syntheses were carried out under various conditions to assess the impact of various factors, particularly the type of iron salts used, the iron salts addition rate, and the reaction time after the addition. Furthermore, to prevent particle oxidation and agglomeration, the MIONs were directly coated with PEG during synthesis. The effect of the *M_n_* of PEG and its concentration in alkaline aqueous medium were also investigated. Table 4 contains the data.

When the iron salts solution was added to the ammonia solution, it darkened quickly and became black and opaque (for samples S2–S11, Table 4, Figure S1—Supplementary Materials). The obtained products were strongly attracted by a permanent neodymium magnet, allowing for simple and effective purification via magnetically assisted sedimentation (Figure S2—Supplementary Materials). The products were in the form of black powder after freeze drying.

**Figure 7:**
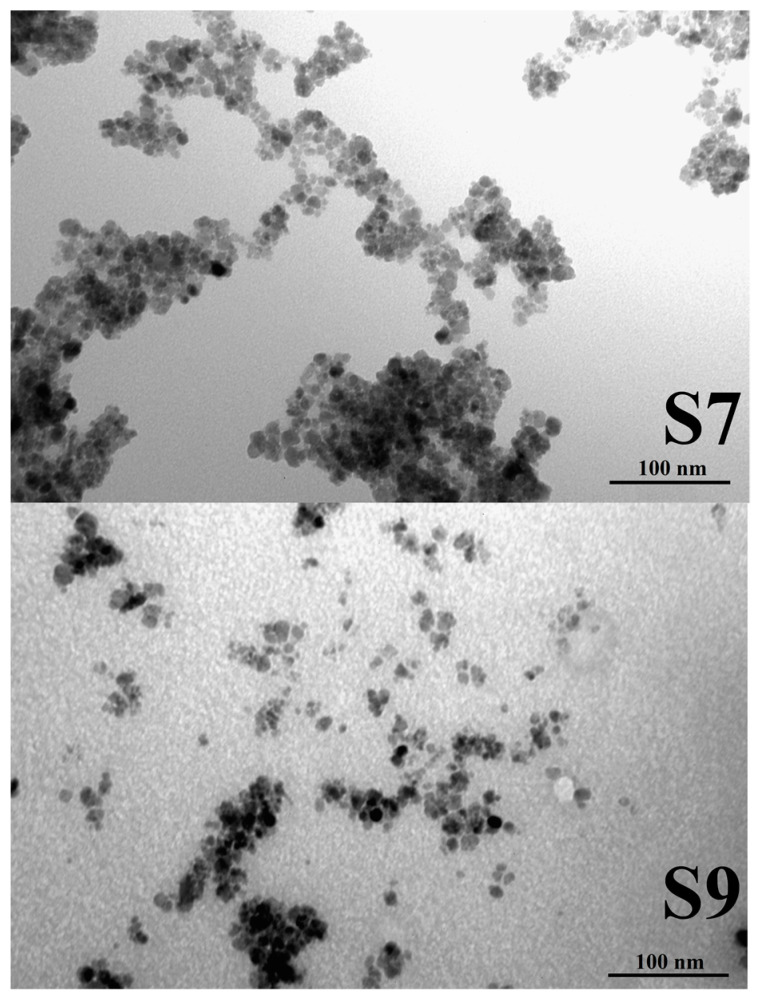
Micrographs of MIONs obtained using the TEM technique.

Interestingly, when Fe_2_(SO_4_)_3_ was used as an iron source (sample S1), the product was rusty and its interaction with the permanent magnet was barely noticeable. It is presumed that this reaction’s product was not magnetite. The nanoparticles’ size and shape were assessed using TEM. According to micrographs (Figure 7), the particles were spherical and homogeneous, with an average size of approximately 9 nm and a narrow dispersity (Table 5). It appears that the investigated factors in the studied range have no effect on the particle shape, size, and size distribution observed by TEM. Dropwise addition of iron salts into a vigorously stirred alkaline solution results in rapid nucleation of Fe_3_O_4_, followed by slow size increase, resulting in nanoscale particles with a homogeneous size and shape.

**Figure 8:**
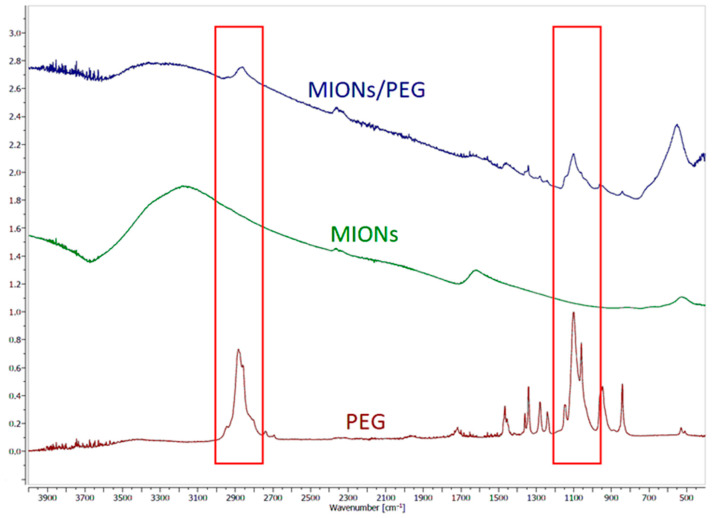
ATR-FTIR spectra of PEG 2000, MIONs and PEG-covered MIONs (sample S8). The red boxes represent the main PEG bands at 2880 cm^−1^ and 1100 cm^−1^, also visible on MIONs/PEG spectrum.

The very small diameter of the resulting nanoparticles can also be attributed to the fact that the coating process was performed directly during the synthesis stage. Following nucleation, the particles are quickly covered by the coating agent’s macromolecule. The process significantly reduces particle growth, agglomeration, and oxidation. The PEG-covering process of the MIONs was investigated using attenuated total reflectance Fourier-transform infrared spectroscopy (ATR-FTIR). Figure 8 depicted the ATR-FTIR spectra.

The MIONs spectrum shows a broad band at 3170 cm^−1^, attributed to the -OH vibrations of absorbed water molecules at the nanoparticles surface and the H-O-H bending band at 1620 cm^−1^. The band at 530 cm^−1^ comes from Fe-O stretching in Fe_3_O_4_. The main bands of PEG, attributed to -CH_2_ stretching and C-O-C stretching, are visible at 2880 cm^−1^ and 1100 cm^−1^, respectively. The other PEG characteristic bands at 1470 cm^−1^ and 1280 cm^−1^ are attributed to -CH_2_ bending. The 950 cm^−1^ band results from out-of-plane -CH bending [49,50,57].

The ATR-FTIR spectrum of the PEG-covered MIONs demonstrates that the main bands corresponding to -CH_2_ and C-O-C stretching vibrations (2880 cm^−1^ and 1100 cm^−1^, respectively) from the PEG molecule are clearly visible. It proves that the nanoparticles were efficiently coated.

The efficient coating has a significant impact on MION stability in aqueous dispersions. Uncoated nanoparticles agglomerate quickly and are therefore unsuitable for medical applications. The stability of the synthesized MIONs was assessed using DLS and visual observation of the nanoparticle sedimentation process over time [58]. Table 4 and Table S3 (Supplementary Materials) contain the results of the DLS and the sedimentation experiments.

The diameter of the particles measured by DLS is typically higher than the diameters observed in TEM. This phenomenon could be explained by the presence of PEG molecules covering the Fe_3_O_4_ magnetic core of the particles, the formation of extended hydrogen bond networks between PEG ethylene groups and the surrounding solvent [57,59]. However, the differences between the mean diameter of the nanoparticles derived from DLS and TEM are too large to be explained only by the aforementioned factors and they are undoubtedly connected with slight agglomeration of the nanoparticles.

In general, when the DLS results are compared to the sedimentation test results, it can be concluded that lower hydrodynamic diameter particles have higher colloidal stability, most likely due to a more effective coating. According to the results of the analysis, the coating efficiency was primarily determined by the coating agent concentration and its *M_n_*, the iron salts mixture addition ratio, and the reaction time.

PEG concentration must be sufficiently high to produce MIONs with acceptable stability [47]. After 2 h, the aqueous suspensions of MIONs synthesized using low PEG concentrations (4 wt.%–8 wt.%) were completely sedimented. Surprisingly, increasing the concentration by up to 30% increased the nanoparticles’ stability while decreasing their hydrodynamic diameter and dispersity index (sample S4, Table 4). Similar behavior was observed for the samples synthesized with PEG 6000 (samples S6 and S7); the optimal PEG concentration was denoted as to be 30%. However, it was discovered that increasing the coating agent concentration up to 36% has a negative effect on particle stability; the sedimentation process was faster and the hydrodynamic diameter was larger with a wider dispersity index (samples S7 and S11). This could be due to the relatively high viscosity of the PEG-containing alkaline medium. Probably, the effectiveness of magnetic stirring was insufficient, and the particles formed larger clusters as a result of extended agglomeration.

The effect of the *M_n_* of PEG was challenging to quantify; the difference between PEG 1000 and PEG 6000 did not appear to be significant. Nonetheless, when PEG 2000 was used at a concentration of 30% (sample S5), the particle stability was severely limited.

The iron salts solution addition ratio into the alkaline medium was optimized in the range of 0.50–0.125 mL/min. The ratio was reduced from 0.50 mL/min to 0.25 mL/min, which appears to slightly improve particle stability (samples S7 and S9), but further reduction to 0.125 mL/min seemed to have no impact.

MIONs were formed almost instantly after the addition of iron salts solution to the alkaline medium, but the covering process took longer. The stability of MIONs in aqueous dispersions was different for samples S8 and S9, which were purified immediately after synthesis and 1 h of mixing, respectively. When the product was purified immediately, the particles were not stable, and the hydrodynamic diameter was 765 nm with a dispersity index of 0.53. After 1 h (sample S9), the diameter and the dispersity index were significantly reduced to 251 nm and 0.32, respectively (Table 4).

The optimal PEG-covered MIONs synthesis conditions were identified using the previously mentioned data. The synthesis required the use of FeCl_3_ and FeSO_4_ as sources of Fe(III) and Fe(II), respectively; the iron salts addition rate was set to 0.25 mL/min, with a reaction time of 1 h. The covering process must be done directly during the synthesis using PEG 6000 at a concentration of 30%. The particles obtained under these conditions had a low diameter of Fe_3_O_4_ crystallites (7.7 nm), sufficient stability in aqueous media, high magnetization, and magnetic properties close to superparamagnetics.

### 2.5. MIONs Magnetic Properties as Hyperthermia-Inducing Devices

#### 2.5.1. VSM Experiment 

**Figure 9:**
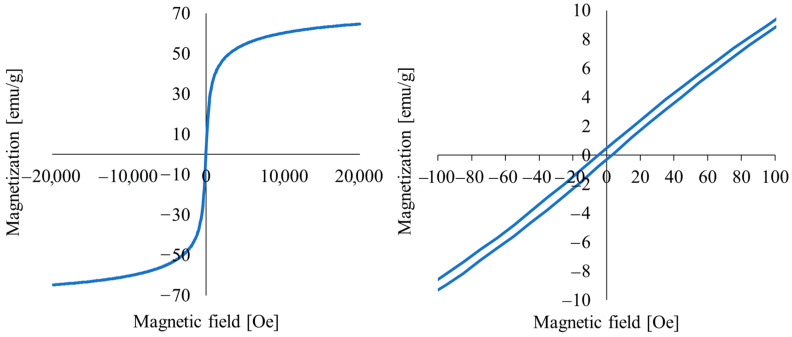
The hysteresis loop of the resulting MIONs (sample S9).

VSM experiments were performed on the selected samples to evaluate their magnetic properties. Figure 9 shows one of the registered hysteresis loops.

MIONs hysteresis loops were S shaped and narrow. The observed *H_c_* and *B_R_* values of the samples were very low (Table 6), indicating that the properties of the obtained nanoparticles were close to superparamagnetic. Because of the small diameter of MIONs, the entire crystallites are single-domain particles that can flip their magnetization due to thermal energy. Thus, the particles exhibit magnetic properties only in the presence of an external magnetic field, preventing the formation of agglomerates due to magnetic attraction between the particles, which is critical for biomedical applications.

According to the VSM data, the *B*_R_ and *H*_c_ values of the S2 sample were slightly higher than the other samples measured. The wider hysteresis loop could be attributed to the higher diameter of the S2 particles (Table 5) as a result of the ineffective coating during the synthesis stage. Superparamagnetic properties are known to disappear as particle diameter increases.

Notably, saturation magnetization was not clearly observed for the measured samples in the experimental external magnetic field range (up to 20,000 Oe). The observed magnetization values for all samples were comparable, as shown in Table 6. However, the magnetization of the obtained MIONs at 20,000 Oe (*M*_20,000 Oe_) and calculated for sample S2, was slightly higher than that for other samples, probably due to wider hysteresis loop and higher diameter of the nanoparticles. Nevertheless, the observed *M*_20,000 Oe_ values for all of the examined samples were relatively high, indicating that they could be used as a heat-generating device in MHT.

#### 2.5.2. Hyperthermia

The amount of energy produced during induction heating of the magnetic nanoparticle suspension is affected by particle size and magnetic properties, as well as AMF frequency (*f*) and amplitude (*H*). Higher values of these parameters correspond to increased heat—the amount of power [W] generated by 1 g of the particles—and this is among the quantitative parameters describing the heating capacity of the nanoparticles. The SAR can be defined in adiabatic conditions, where all of the generated heat is transferred into temperature increase, by the equation:

(1) $$SAR=\frac{P}{m}=\frac{c\frac{∆T}{∆t}}{m}\left[\frac{W}{g}\right]$$

where *P* is the heating power of the nanoparticles [W], *m* is the mass of nanoparticles [g], *c* is the sample heat capacity [J/K], and Δ*T/*Δ*t* is the slope of temperature increase per time increment [K/s] [60,61,62].

In non-adiabatic conditions, where most SAR evaluations are performed, heat loss is inevitable and should be taken into account:

(2) $$P=c\frac{∆T}{∆t}+L_{h}\left(T-T_{0}\right)$$

where *L_h_* [W/K] quantifies heat loss of the system proportionally to the difference between sample and surrounding temperatures, *T* and *T*_0_ [K], respectively [60]. 

The equation assumes that heat loss is linearly proportional to the temperature difference between the sample and the surrounding environment. Furthermore, when the sample temperature reaches the specific temperature, heat loss will equal the heating power of the nanoparticles, and the heating curve will reach a plateau [60]. 

Notably, the SAR is strongly dependent on the AMF parameters, making comparisons of SAR values measured with different devices difficult. As a result, the intrinsic loss power (ILP) parameter has been calculated using the following formula:

(3) $$ILP=\frac{SAR}{{fH}^{2}}\left[\frac{Hm^{2}}{kg}\right]$$

Biosafety is among the most critical issues associated with all biomedical devices. In general, an increase in *f* and *H* can be advantageous due to an increase in SAR value. However, if the AMF is too strong, it can generate too much eddy current in the tissues, resulting in potentially harmful, non-specific heat generation and neural stimulation [60]. To address the biosafety requirements, the maximum AMF strength is described as *Hf* ≤ 5 × 10^9^ [A/(m·s)] [32,63].

**Figure 10:**
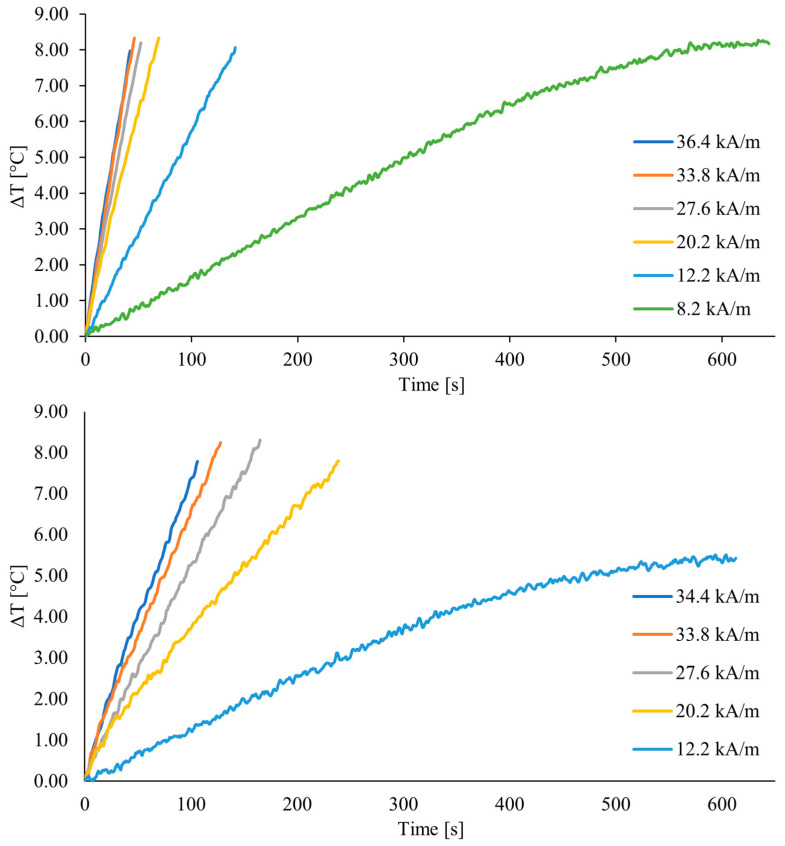
Heating curves for MION suspensions at 5 mg/mL (top) and 2 mg/mL (bottom) (sample S9).

To evaluate the heating potential of the obtained MIONs, the SAR and ILP values were determined using induction heating device. The heating curves of 5 and 2 mg/mL MION suspensions (sample S9), recorded under various AMF amplitudes, are shown in Figure 10.

The SAR and ILP parameters were calculated using the corrected slope method based on the recorded heating (Figure 10) and cooling curves [60]. The parameters are presented in Table 7. 

The ideal temperature for hyperthermia treatment is 41−45 °C, which corresponds to ΔT of 4.4−8.4 °C (an increase from 36.6 °C). According to the heating curves (Figure 10), the eligible temperature increase (up to 8.4 °C) was observed in a short time period (˂5 min) for the majority of the tested *H* values and MION concentrations. Furthermore, the relationship between ΔT and time appears to be linear in most cases. Different behavior was observed for low *H* values—8.2 kA/m (5 mg/mL) and 12.2 kA/m (2 mg/mL). The heating time was significantly longer and the plateau phase was clearly observed after 10 min (600 s); the maximum ΔT reached 8.2 °C and 5.4 °C for 8.2 kA/m (5 mg/mL) and 12.2 kA/m (2 mg/mL), respectively.

It was discovered that as *H* value increased, the recorded heating curve slopes and calculated SAR values increased significantly. Nonetheless, taking into account the *Hf* limitations for biomedical devices, the maximum *H* value for the examined *f* (approximately 360 kHz) was 12.2 kA/m. As a result, the optimal MION concentration was designated as 5 mg/mL, because for the lower concentration (2 mg/mL), the time needed for a minimal desirable increase in temperature was significantly longer. Moreover, the *plateau* phase was clearly observed for ΔT of 5.4 °C (T = 42.0 °C). The heating curve was non-linear, making it difficult to precisely control the temperature. Thus, it was concluded that when the MION concentration was 2 mg/mL, there were not enough particles to generate a sufficient amount of heat to provide stable and well-controlled hyperthermia conditions, under applicable AMF (360 kHz, 12.2 kA/m). On the other hand, the heating abilities of a 5 mg/mL suspension under these conditions were suitable for MHT; the heating curve was linear and the desirable temperature was reached within short time period. Therefore, the further increase in the concentration was deemed redundant. The SAR and ILP values were satisfactory—67.6 W/g and 1.261 nHm2/kg, respectively—and comparable to similar magnetic nanoparticles described in the literature [32].

### 2.6. Drug Release Studies and Hydrolytic Degradation of the PCLA Hydrogels

**Figure 11:**
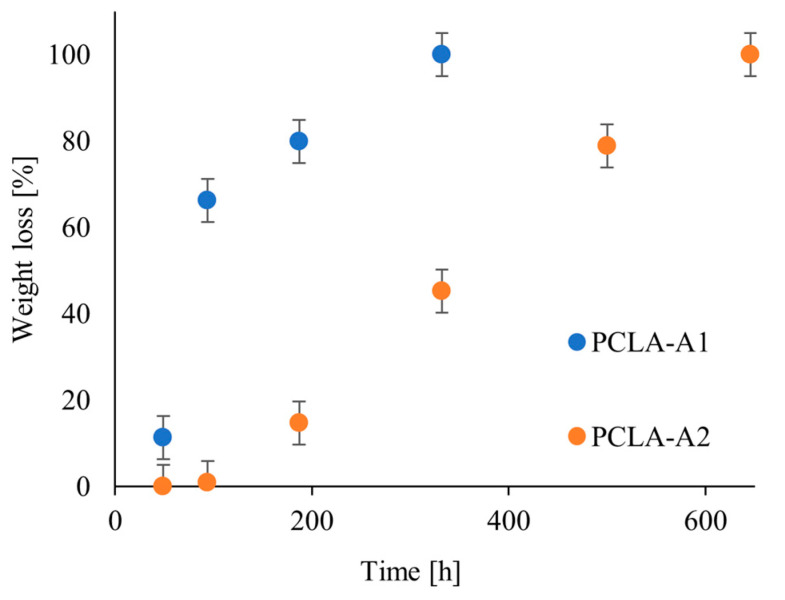
Hydrolytic degradation profiles of the PCLA-A1 and PCLA-A2 hydrogels at 25 wt.%.

The in vitro hydrolytic degradation and drug release profiles of the obtained thermosensitive hydrogels were investigated to determine their potential as antitumor DDSs. Figure 11 shows the hydrolytic degradation profiles of the PCLA-A1 and PCLA-A2 hydrogels (25 wt.%)

According to the data, the PCLA-A1 hydrogel degradation rate is faster than the PCLA-A2 hydrogel degradation rate; the matrices were fully decomposed after 332 and 646 h, respectively. The difference in the structure of the copolymer’s hydrophobic chains explains this fact. The PCLA-A1 copolymer contained more LA domains (the LA/PEG ratio was 1.1 [m/m], compared to 0.2 for PCLA-A2) and fewer CL domains (the CL/PEG ratio was 1.5 [m/m], compared to 2.1 for PCLA-A2, Table 2). As a result, the PCLA-A1 was more hydrophilic, allowing water molecules to penetrate the matrix more easily, and the hydrogel based on this copolymer decomposed more quickly.

Furthermore, the degradation of PCLA-A1 appears to occur in phases. The degradation rate increased significantly between the 49 h and 94 h of the experiment. It is assumed that after 49 h, the hydrogel’s intensive swelling process weakened the interactions between copolymer chains, making the matrix more flexible and easier to penetrate for water. As a result, the amphiphilic PCLA macromolecules in the matrix could be eluted, resulting in the hydrogel decomposition. This effect was barely noticeable for the PCLA-A2 sample, owing to its higher hydrophobicity and different hydrophobic chain structure. Furthermore, the degradation rate of the PCLA-A2 hydrogel appears to be constant throughout the experiment, which may be advantageous for drug release properties.

**Figure 12:**
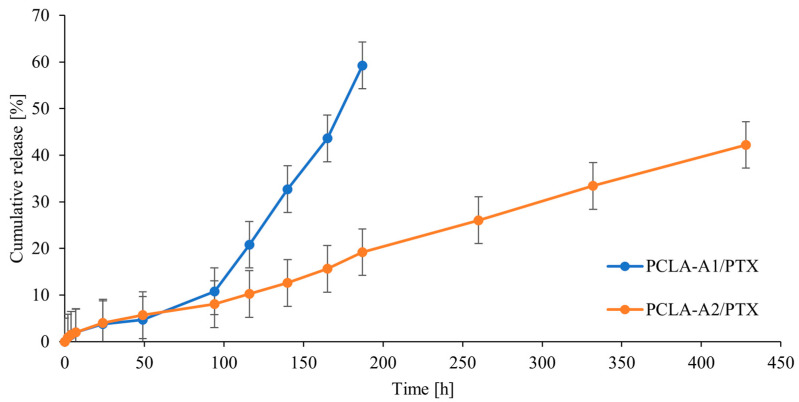
Drug release profiles of the PCLA-A1/PTX and PCLA-A2/PTX hydrogels.

For in vitro drug release studies, 2.5 mg of PTX was incorporated into the PCLA-A1 and PCLA-A2 hydrogels; the samples were denoted as PCLA-A1/PTX and PCLA-A2/PTX, respectively. The drug release profiles (Figure 12) were fitted to zero-order, first-order, Higuchi and Korsmeyer–Peppas models. Table 8 displays the results.

The drug release profiles of the examined hydrogels were prolonged and controlled. PTX release from the PCLA-A1/PTX hydrogel can be divided into two phases: the initial release rate was relatively low, 4.7% of the drug was continuously released after 49 h. The release kinetics could not be determined unambiguously using comparable zero and first order R^2^ values, calculated for this phase. Despite this, the high R^2^ for the Higuchi model, as well as the great fitting for Korsmeyer–Peppas with a *n* value of 0.517, indicate that the diffusional release mechanism is dominant. The hydrogel degradation rate was relatively low at this stage and the hydrophobic drug was most likely eluted from the surface and outer layers of the matrix. Subsequently, between 94 h and 187 h of the experiment, the drug release profile changed due to the switch of the release mechanism. According to mathematical models, in the second phase of the release, the *n* value from the Korsmeyer–Peppas model suggested the Super Case II release mechanism, leading to zero-order kinetics. The release rate was primarily determined by the decomposition of the hydrogel matrix. A high R^2^ value for this model confirmed that they displayed close to zero-order kinetics.

The obtained PCLA-A1/PTX drug release profile suits the hydrolytic degradation data precisely. When the hydrogel decomposition rate increased significantly due to the matrix swelling, the mechanism switched from predominantly diffusional to erosive. A similar phenomenon is believed to have occurred for the PCLA-A2/PTX hydrogel, but the effect was barely noticeable; the two phases of the drug release could not be clearly distinguished. Because of the matrix’s higher hydrophobicity and very low PTX water solubility, the drug release profile was prolonged and well controlled for up to 428 h. According to the mathematical models, drug release displayed close to zero-order kinetics (R^2^ = 0.9944, Table 8). However, because the R^2^ values for zero- and first-order models were comparable, determining the exact release kinetic studies was impossible. When the diffusion and erosion processes occurred concurrently, the release mechanism was determined to be anomalous (*n* = 0.690, R^2^ = 0.9761). 

According to the literature, the observed drug release from PCLA thermosensitive hydrogels was controlled and prolonged for up to 30 days. In general, the release kinetics were close to first order, and matrix decomposition was observed during the experiments [3,64]. Li and coworkers [23] obtained the PCLA hydrogel as injectable flurbiprofen DDS. The drug release profile for 25 wt.% hydrogel was prolonged; 80% of the drug was released in 24 days. However, the kinetic seemed to be close to first order; the drug release mechanism was not determined. Similar outcomes were published by Sharma et al. [65]. The PCLA hydrogel was used for ketoprofen delivery. The in vitro drug release was shorter (120 h) and displayed close to first-order kinetics. According to the Korsmeyer–Peppas model, the drug release mechanism was found to be anomalous, combining diffusion and erosion processes. Therefore, it is believed that the drug release behavior of the PCLA-based hydrogels is strongly dependent on drug solubility. The results obtained for tacrolimus [66] and PTX [67] DDSs, based on methoxy-PEG-P(CL-*co*-LA) and sulfamethazine-modified PCLA copolymers, appear to confirm that statement. The drug release profile from the PCLA hydrogels for hydrophobic and insoluble drugs was found display close to zero-order kinetics. As a result, the PCLA-A2/PTX hydrogel was selected as the most promising candidate for further research.

**Figure 13:**
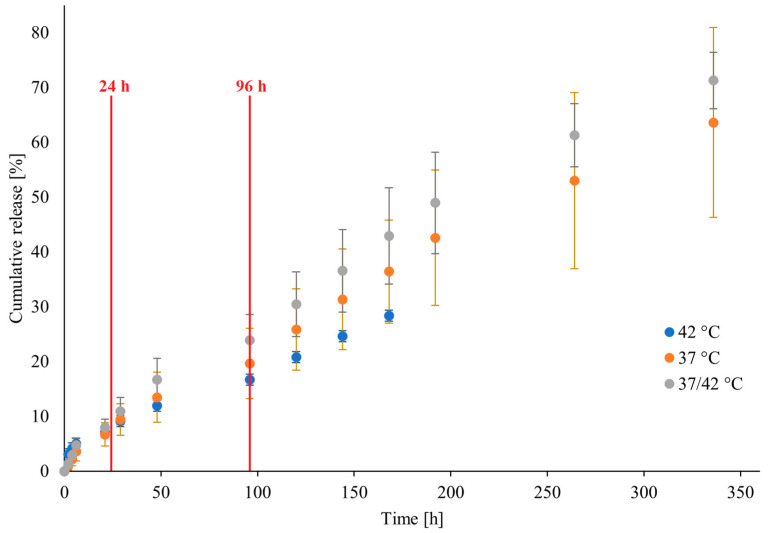
Drug release profiles recorded in 42 °C, 37 °C and pulsatile temperature increase conditions (37/42 °C). Red vertical bars represent the simulated hyperthermia events. Each sample was prepared in triplicate. The data points were presented as cumulative release ± SD (n = 3).

The experiments In hyperthermia-simulating conditions were carried out to evaluate the effect of elevated temperature on the drug release profile; the temperature was periodically increased to 42 °C after 24 h and 94 h for 60 min. For comparison, the drug release experiments at constant temperatures (37 °C and 42 °C) were carried out concurrently. The achieved PTX release profiles (Figure 13) were displayed for 336 h. Following that, the profiles reached a plateau. Furthermore, after 336 h, the matrices were nearly fully decomposed.

First and foremost, the obtained data show that antitumor drug release from the PCLA-A2/PTX/MIONs hydrogels is sustained and well controlled under all conditions tested. Analyzing the effect of MIONs on the drug release, no significant differences were observed when comparing 37 °C (sample with 5 mg/mL MIONs, Figure 13) and PCLA-A2 (without MIONs, Figure 12) profiles characteristics. As a result, the MIONs had no effect on the drug release profiles.

The effect of hyperthermia on the PTX release profiles from the PCLA-A2/PTX/MIONs hydrogel appears to be negligible. Within 168 h, there are no discernible differences between the drug release profiles at 37 °C and 42 °C. Furthermore, the increase in cumulative release between 21 h and 29 h for 37 °C and 37/42 °C profiles was comparable; the 60-min hyperthermia simulation performed between those data points had no effect on the drug release rate. The effects of the hyperthermia simulation after 96 h are consistent. Figure 13 shows that regardless of the experimental conditions, all of the recorded drug release profiles are almost identical. This phenomenon could be probably explained by the nature of the PCLA-A2 hydrogel. The temperatures of 37 °C and 42 °C were discovered in a gel window of the PCLA-A2 hydrogel. Furthermore, the recorded G’ and G” values for these two temperatures are comparable. As a result, for PTX release profiles, the difference in hydrogel properties between these two temperatures appears to be insignificant. Similar results have previously been observed in the literature [32,68]. According to the data, elevated temperature (approximately 42 °C) may increase the rate of drug release, but this effect is not always observed; it depends on the type of hydrogel and its properties, as well as the nature of the drug.

The data were fitted to mathematical models to assess the drug release kinetics and mechanism. Table 9 shows the results.

Since the R^2^ values calculated for the sample are high, the drug release profiles were denoted to be close to the zero-order model. However, the kinetics cannot be described unambiguously; the R^2^ for zero- and first-order models was identical. According to the drug release mechanism considerations, all of the examined profiles match the Higuchi model perfectly, implying a diffusional release mechanism. The results of the Korsmeyer–Peppas model differ slightly. The *n* values for 37 °C and 37/42 °C were 0.796 and 0.712, respectively, indicating an anomalous drug release mechanism. The mechanism consists of drug diffusion and hydrogel erosion processes, resulting in zero-order kinetics. The *n* value for the 42 °C profile was 0.524, indicating that the mechanism was primarily diffusional. Interestingly, the profile at 42 °C appears to be identical to other ones during the first 7 days of the experiment (with anomalous release mechanisms). It is assumed that the mechanism was predominantly diffusional at the beginning of the experiment; the drug was eluted from the surface and outer layers of the hydrogel. As a consequence of the matrix erosion, the mechanism switched to anomalous, resulting in closer to zero-order kinetics. These findings corroborate previous findings for PCLA-A1 and PCLA-A2 samples (Figure 12). Nonetheless, according to both profiles, at 37 °C and 37/42 °C, almost identical R^2^ and *n* values for all mathematical models used indicate that hyperthermia has no significant effect on PTX release from the obtained PCLA-A2/PTX/MIONs hydrogel. As a result, the hydrogel appears to be a promising LDDS for novel chemotherapy combined with hyperthermia.

## 3. Materials and Methods

### 3.1. Materials

ε-Caprolactone (2-Oxepanone, CL, 97%, Aldrich, Poznan, Poland), *rac*-lactide (3,6-dimethyl-1,4-dioxane-2,5-dione, LA, ≥ 96%, Sigma-Aldrich, Poznan, Poland), zirconium(IV) acetylacetonate (tetrakis(2,4-pentanedionato)zirconium(IV), Zr(acac)_4_, 97%, Aldrich, Poznan, Poland), poly(ethylene glycol) 1000 (PEG 1000, *M_n_* = 1000 g/mol, pure, Sigma-Aldrich, Poznan, Poland), poly(ethylene glycol) 1500 (PEG 1500, *M_n_* = 1500 g/mol, pure, Sigma, Poznan, Poland), poly(ethylene glycol) 2000, (PEG 2000, *M_n_* = 2000 g/mol, pure, TCI, Zwijndrecht, Belgium), poly(ethylene glycol) 6000, (PEG 6000, *M_n_* = 6000 g/mol, Sigma-Aldrich, Poznan, Poland), iron(III) chloride hexahydrate (FeCl_3_ × 6 H_2_O, ≥ 99%, Sigma-Aldrich, Poznan, Poland), iron(III) sulfate hydrate (Fe_2_(SO_4_)_3_ × H_2_O, pure, ChemPur, Piekary Slaskie, Poland), iron(II) sulfate heptahydrate (FeSO_4_ × 7 H_2_O, ≥ 99.0%, Sigma-Aldrich, Poznan, Poland), ammonia solution 25% (NH_3_ × H_2_O, pure, ChemPur, Gliwice, Poland) dichloromethane (DCM, CH_2_Cl_2_, ≥ 99.8%, POCH, Gliwice, Poland), toluene anhydrous (Acros Organics, 99.8%, Extra Dry, Gdansk, Poland), Dulbecco’s Modified Eagle Medium (DMEM, Thermo Fisher Scientific, Warsaw, Poland), phosphate-buffered saline (PBS, pH 7.40 ± 0.05, ChemPur, Piekary Slaskie, Poland), phosphate-buffered saline (PBS, GIBCO, Dublin, Ireland), Cremophor EL (Polyoxyl-35 castor oil, pure, Sigma-Aldrich, Poznan, Poland), paclitaxel (PTX, pure, Sigma-Aldrich, Poznan, Poland), acetonitrile (ACN, gradient grade for liquid chromatography, Merck, Darmstadt, Germany), methanol (MeOH, for liquid chromatography, Merck, Darmstadt, Germany), and trifluoroacetic acid (TFA, 99%, Sigma-Aldrich, Poznan, Poland). PEG 1500 used for copolymer synthesis was heated in 80 °C for 2 h in vacuum to remove water residues. Other reagents were used as received.

### 3.2. NMR Data

The ^1^H NMR spectrum of CL, LA and PEG copolymer: 1.40 ppm (-CO-CH_2_-CH_2_-**CH_2_**-CH_2_-CH_2_-O-), 1.48 ppm (-CO-CH(**CH_3_**)-O-), 1.64 ppm (-CO-CH_2_-**CH_2_**-CH_2_-**CH_2_**-CH_2_-O-), 2.30 ppm (-CO-**CH_2_**-CH_2_-CH_2_-CH_2_-CH_2_-O-) in -CO-*Cap-**Cap-*** sequences, 2.38 ppm (-CO-**CH_2_**-CH_2_-CH_2_-CH_2_-CH_2_-O-) in -CO-*Lac-**Cap***- sequences, 3.63 ppm (-**CH_2_**-**CH_2_**-O-), 4.05 ppm (-CO-CH_2_-CH_2_-CH_2_-CH_2_-**CH_2_**-O-) in -CO-***Cap****-Cap-* sequences, 4.13 ppm (-CO-CH_2_-CH_2_-CH_2_-CH_2_-**CH_2_**-O-) in -CO-***Cap****-Lac* sequences, 4.21 ppm (-CH_2_-CH_2_-O-CH_2_-**CH_2_**-O-*Cap*-), 4.27 ppm (-CH_2_-CH_2_-O-CH_2_-**CH_2_**-O-*Lac*-), and 5.04 ppm (-CO-**CH**(CH_3_)-O-).

The ^13^C NMR spectrum of CL, LA and PEG copolymer: 17.02 ppm (-CO-CH(**CH_3_**)-O-), 20.49 ppm (-CO-CH(**CH_3_**)-OH) end groups, 24.62 ppm (-CO-CH_2_-**CH**_2_-CH_2_-CH_2_-CH_2_-O-), 25.57 ppm (-CO-CH_2_-CH_2_-**CH_2_**-CH_2_-CH_2_-O-), 28.39 ppm (-CO-CH_2_-CH_2_-CH_2_-**CH_2_**-CH_2_-O-), 32.36 ppm (-CO-CH_2_-CH_2_-**CH_2_-CH_2_**-CH_2_-OH) end groups, 34.16 ppm (-CO-**CH_2_**-CH_2_-CH_2_-CH_2_-CH_2_-O-), 62.57 ppm (-CO-CH_2_-CH_2_-CH_2_-CH_2_-**CH_2_**-OH) end groups, 63.49 ppm (-CH_2_-CH_2_-O-CH_2_-**CH_2_**-O-CO-), 64.18 ppm (-CO-CH_2_-CH_2_-CH_2_-CH_2_-**CH_2_**-O-), 66.77 ppm (-CO-**CH**(CH_3_)-OH) end groups, 69.21 ppm (-CO-**CH**(CH_3_)-O-) and (-CH_2_-CH_2_-O-**CH_2_**-CH_2_-O-CO-), 70.60 ppm (-**CH_2_**-**CH_2_**-O-), 170.90 ppm (-**C**O-CH(CH_3_)-O-) in lactyl units (*L*) in -*Cap-**L**-Cap-* sequences, and 173.57 ppm (-**C**O-CH_2_-CH_2_-CH_2_-CH_2_-CH_2_-O-).

### 3.3. Copolymer Synthesis

Following the described procedure, the PCLA copolymers were synthesized in a ROP process carried out in bulk conditions. Amounts of 1.10 g CL, 1.10 g LA, and 1.00 g PEG 1500 were placed in a 10 mL glass ampoule under argon. The ampoule was then tightly sealed after the calculated amount of catalyst (Zr(acac)4) was added (as an anhydrous toluene solution). The sample was immersed in an oil bath for a predetermined period of time. The post-reaction mixture was transferred to a crystallizer and carefully washed with hot, distilled water (approximately 70 °C) after the synthesis. The precipitated, purified product was collected and vacuum dried in room temperature for 48 h. Prior to use, the copolymers were stored at -26 °C. All of the other PCLA copolymers were synthesized in the same way. The initial amount of PEG 1500 used in the larger-scale syntheses was 3.50 g.

### 3.4. MIONs Synthesis

Iron oxide nanoparticles were synthesized by the coprecipitation method [48]. In the first step, the aqueous solution of Fe^3+^ (6.6 mmol) and Fe^2+^ (3.3 mmol) was prepared by dissolving the calculated amounts of ferrous and ferric inorganic salts, namely FeSO_4_·7 H_2_O and FeCl_3_·6 H_2_O (or Fe_2_(SO_4_)_3_·H_2_O) in 7.5 mL of distilled and deoxygenated water. The oxygen was removed from the water after 45 min of argon purging. The Fe^3+^/Fe^2+^ molar ratio was constant and kept at 2:1. Under vigorous magnetic stirring (2000 rpm), the obtained solution was added dropwise to 75 mL of deoxygenated ammonia (0.8 mol/L) containing 30% PEG 6000 (PEG 6000 was used as a stabilizer and a nanoparticle coating agent). The mixture was then stirred for 1 h to complete the precipitation process and crystal growth. To prevent oxidation, all synthesis procedures were carried out in an inert gas atmosphere. The nanoparticles were separated from the reaction mixture by sedimentation with a strong neodymium magnet and carefully washed several times with deoxygenated water to neutral pH before being freeze dried. All of the MION samples were synthesized using the same procedure, but with different Fe(III) salts, iron salts solution addition rate, PEG molecular weight, and concentration. The optimal reaction conditions were determined based on the data collected.

### 3.5. Hydrolytic Degradation and Drug Release Studies

The in vitro drug release studies were performed according to membranelles method [16]. The matrices were first prepared by dissolving the calculated amount of the selected PCLA copolymer in MION suspension to obtain 25 wt.% solutions. To obtain a homogeneous liquid, predetermined nanoparticle concentration suspensions were sonicated for 15 min. PTX were then added to the matrix. 1.0 mL of the hydrogel containing 2.5 mg of PTX and 5.0 mg of MIONs was injected into a 5 mL vial using a 22 gauge needle. The samples without MIONs were prepared in the same manner. The samples were kept at 37 °C for 15 min to form opaque and semi-solid gels. The gels were then immersed in 5.0 mL of preheated Cremophor EL in PBS (pH = 7.4) solution and gently shaken (70 rpm) at 37 °C. The release medium was withdrawn after predetermined time intervals and replaced with 5.0 mL of fresh, preheated solution. Prior to HPLC analysis, the collected medium samples were stored at -26 °C. The PCLA-A2/PTX/MIONs hydrogels were obtained using the aforementioned procedure to assess the effect of hyperthermia treatment on the drug release profiles. Following that, the in vitro drug release experiments were carried out under various conditions, with each sample prepared in triplicate. The experiment was conducted in 37 °C for 21 days to simulate hyperthermia conditions. The temperature was temporarily raised to 42 °C for 1 h after predetermined time periods (24 and 96 h). For comparison, release experiments at constant temperatures of 37 °C (21 days) and 42 °C (7 days) were also carried out.

The hydrolytic degradation assay was carried out concurrently with the drug release studies, as previously described [16,69]. In brief, 1.0 mL of the 25 wt.% hydrogel was injected into a 5 mL vial and allowed to form a semi-solid gel for 15 min at 37 °C. Following that, the gel was immersed in 5.0 mL of preheated medium (1 wt.% Cremophor EL solution in PBS (pH = 7.4)) and shaken (70 rpm) at 37 °C. After 49 h, 94 h, 187 h, 332 h, 500 h, and 646 h, the medium was completely replaced with a fresh solution. The collected samples were vacuum dried. The residues were weighed, and the resulting masses were used to calculate the weight loss of the hydrogels over time using the formula below [69]:

(4) $$W=W_{d}-W_{o}-W_{p}$$

where *W* is the degraded copolymer weight, *W_d_* is the weight of the residue-containing vial after vacuum drying, *W_o_* is the mass of the original vial and *W_p_* is the weight of phosphate salts and Cremophor EL residues from the release medium.

### 3.6. Measurements

#### 3.6.1. Structural Analysis

The structure of the synthesized PCLA copolymers, *M_n_* and monomer conversion were evaluated using the ^1^H and ^13^C NMR techniques. The spectra were recorded on a Agilent Technologies 400 MHz (Santa Clara, CA, USA) spectrometer.

The *M_n_* of the obtained copolymers as well as the average number of caproyl (*n_CL_*) and lactydyl (*n_LA_)* units in the copolymeric chain were estimated using following formulas [44,70]:

(5) $$n_{CL}=\frac{I_{E}}{I_{F}}\cdot 2\left(n_{EG}-1\right)$$

(6) $$n_{LA}=\frac{I_{A}}{I_{F}}\cdot 2\left(n_{EG}-1\right)$$

(7) $$M_{n}=114n_{CL}+144n_{LA}+44n_{EG}$$

where *I_E_* is the integral intensity of the protons adjacent to the α carbon atom of caproyl units (-CO-**CH_2_**-CH_2_-CH_2_-CH_2_-CH_2_-O-) in the PCLA polymeric chain, *I_A_* corresponds to methine protons of lactyl units (-CO-**CH**(CH_3_)-O-), *I_F_* is the integral intensity of methylene protons of PEG (-**CH_2_**-**CH_2_**-O-) and *n_EG_* is the average number of ethylene glycol mers in the PEG molecule.

The average length of the -*Cap*-*Cap*- sequence in the synthetized copolymers was calculated from ^1^H NMR spectra using the method described by Jiang et al. [44]. For the PCL/PLA blocks, where the first mer connected to terminal hydroxy group of PEG was LA, the average length of the -*Cap*-*Cap*- sequence was signed as *L_-EG-Lac-_*_._ When the first mer adjacent to EG group is CL, the -*Cap*-*Cap*- sequence average length was denoted as *L_-EG-Cap-_*. The aforementioned parameters and the total average -*Cap*-*Cap*- sequence length (*L_Cap_*) in the polymeric chain were calculated following the equations:

(8) $$L_{-EG-Lac-}=\frac{I_{E_{-Cap-Cap-}}}{I_{E_{-Lac-Cap-}}}+1$$

(9) $$L_{-EG-Cap-}=\frac{\left(\frac{I_{E_{-Cap-Cap-}}}{I_{E_{-Lac-Cap-}}}+1\right)\cdot \frac{n_{CL}}{2}}{\left(\frac{I_{E_{-Cap-Cap-}}}{I_{E_{-Lac-Cap-}}}+1\right)+\frac{n_{CL}}{2}}$$

(10) $$L_{Cap}=\frac{{I_{H_{-EG-Lac-}}L}_{-EG-Lac-}+{I_{H_{-EG-Cap-}}L}_{-EG-Cap-}}{I_{H_{-EG-Cap-}}+I_{H_{-EG-Lac-}}}$$

where $I_{E_{-Cap-Cap-}}$ and $I_{E_{-Lac-Cap-}}$ correspond to integral intensities of methylene protons adjacent to the α carbon atom in CL mers in -CO-*Cap-**Cap***- and -CO-*Lac-**Cap***- sequences, respectively. $I_{H_{-EG-Lac-}}$ is the integral intensity of a signal from methylene protons of EG in -EG-*Lac*- sequences (-CH_2_-CH_2_-O-CH_2_-**CH_2_**-O-*Lac*-), and $I_{H_{-EG-Cap-}}$ comes from analogous protons in -EG-*Cap*- sequences (-CH_2_-CH_2_-O-CH_2_-**CH_2_**-O-*Cap*-).

CL conversion was estimated using the ^1^H NMR spectra of the post-reaction mixture, according to the following formula:

(11) $$convCL=\frac{I_{E}}{I_{E}+I_{mono}}$$

where *I_E_* and *I_mono_* are the integral intensities of protons adjacent to carbon atoms in copolymeric chains and monomer molecules, respectively.

The *M_n_* and *Đ* index values of the synthesized copolymers were determined using the GPC technique. The measurements were carried out on a Malvern Viscotek GPCMax TDA 305 (Malvern Panalytical, Malvern, United Kingdom) chromatograph equipped with a Jordi Gel DVB mixed bed column (Jordi Labs, Mansfield, MA, USA). The mobile phase flow (DCM) was set to 1.0 mL/min, and the column temperature was set to 30 °C. The system was calibrated using polystyrene standards.

#### 3.6.2. Rheological Studies

The rheological properties of the obtained thermosensitive hydrogels were determined using rotational rheometer ARES (TA Instruments, New Castle, Delaware, USA), according to previously described procedure [16]. The 25 wt.% hydrogel was formed by dissolving a calculated amount of the PCLA copolymer in distilled water and placing it between two parallel round plates with a 25 mm diameter and 0.3 mm gap. The G’ and G” were measured in the temperature range of 25–55 °C. The deformation value (*γ*) was set as 0.6%, the heating rate was 1 °C/min with 1 °C sampling.

#### 3.6.3. Sol–Gel–Sol Transition

The tube-inverting method was used to determine the hydrogels phase transition temperatures [6,16]. For LCST and UCST determination, the calculated amount of the PCLA copolymer was placed in a 5 mL glass vial (1 cm inner diameter) and dissolved in 1.00 mL of distilled water to obtain 25 wt.% solution. The mixture was heated to 50 °C, shaken, and placed in a 4 °C refrigerator to aid in homogenization and dissolution. The obtained hydrogel was then thermostated; the temperature was gradually increased (1 °C step; approximately 1 min of heating and 2 min for temperature equlibration) between 25 °C and 55 °C. The sol–gel–sol phase transitions were visually monitored. The state was designated as “gel” when no flow was observed in the inverted glass vial within 15 s.

#### 3.6.4. Cytotoxicity and Genotoxicity Assay

The cytotoxicity of the obtained copolymers was assessed by neutral red uptake test (NRU), according to ISO guidelines [16,54]. Briefly, the BALB/c 3T3 clone A31 mammalian cells (mouse embryonic fibroblasts from American Type Culture Collection, Manassas, VA, USA) were seeded in 96-well microplates and immersed in DMEM medium, supplemented with 10% of calf bovine serum, 100 IU/mL penicillin and 0.1 mg/mL streptomycin, and subsequently incubated under specific conditions (5% CO_2_, 37 °C, > 90% humidity). After 24 h, the culture medium was replaced by 1 mg/mL copolymer extract, prepared by incubation of the tested material in the medium with reduced bovine serum concentration (5%). Cells were treated with four dilutions of each extract in a two-fold dilution series for 24 h (three data points for each one). In the next step, the treated medium was discarded, the cells were washed with fresh PBS and immersed in neutral red medium for 2 h. Following that, the medium was removed, the cells were washed with PBS, and they were treated with a desorbing fixative (ethanol and acetic acid water solution). The amount of dye absorbed was determined colorimetrically (540 nm). As positive and negative controls, latex and polyethylene foil were used. The percentage of viable cells in each well was determined by comparing its OD540 value to the mean value obtained for untreated cells (incubated in the same conditions with fresh culture medium). If the percentage of viable cells did not fall below 70%, the sample was considered to be non-cytotoxic.

The genotoxicity of the synthesized copolymers was evaluated using *umu* test, according to the suitable guidelines [16,55]. The test was performed using *Salmonella typhimurium* TA1535/pSK1002 as the test organism, producing β-galactosidase in response to DNA damage. The secreted enzyme can convert a colorless substrate into a yellow product (ortho-nitrophenyl-β-galactoside) that can be colorimetrically quantified at 420 nm. The *umu* test was made with and without metabolic activation (S9 liver fraction, Xenometrics, Stilwell, KS, USA), using 96-well plates. Distilled and sterilized water was used as the negative control, and 2-aminoanthracene and 4-nitroquinoline N-oxide were used as positive controls. The PCLA extracts were prepared by incubation of the copolymers in PBS at 37 °C for 24 h with shaking. All samples were tested in two-fold dilution series (three concentrations, three points of date for each one). Clear PBS, treated in the same way as all samples, was tested as a solvent control. The samples were compared to a negative control and the IR—the β-galactosidase activity ratio of tested sample in comparison to the negative control—was calculated. When the IR was <1.5, the sample was considered as non-genotoxic.

#### 3.6.5. TEM and DLS Analysis

The size and shape of MIONs were determined using TEM. MION suspensions in diluted water were air dried on Formvar-coated nickel grid. Under 80 kV accelerating voltage, the measurements were taken on a JEM 1400 (JEOL, Tokyo, Japan) microscope outfitted with an 11 Megapixel MORADA G2 TEM camera (EMSIS GmbH, Muenster, Germany). Using ImageJ software (version 1.53k), the mean and median diameters of MIONs were calculated from 100 randomly chosen particles.

The hydrodynamic diameter and zeta potential of MIONs were measured using the Zetasizer Nano ZS DLS apparatus (Malvern, United Kingdom). The measurement angle was 173° and the excitation wavelength was 633 nm (He-Ne laser, power = 5 W). Before measuring, nanoparticles were suspended in water and sonicated for a few minutes. The zeta potential was determined using a standard dip cell with platinum electrodes. All experiments were carried out in four replications at 25 °C.

#### 3.6.6. PEG-Coating Efficiency and MIONs Aqueous Dispersions Stability Analysis

The formation of PEG coating on MIONs surface was investigated using ATR-FTIR analysis. The IR spectra (4000 cm^−1^–400 cm^−1^) were recorded on a FT-IR IRAffinity-1S (Shimadzu, Kyoto, Japan) spectrometer equipped with Quest ATR (Specac, Orpington, United Kingdom) accessory. The number of scans was set to be 30 for each sample.

The sedimentation test was used to assess the colloidal stability of MIONs [58]. An amount of 15 mg of nanoparticles were placed in a glass vial (1 cm inner diameter), suspended in 3 mL of water and sonicated for 10 min. The sedimentation process was observed visually for 7 days.

#### 3.6.7. Magnetic Properties of MIONs

The magnetic properties of MIONs, namely *H_c_*, *B_R_* and *M*_20,000_ *Oe*, were measured using the VSM technique. The magnetic hysteresis loops were recorded on Physical Property Materials System (PPMS) by Quantum Design (Darmstadt, Germany) with VSM Option at room temperature in the range of magnetic field ± 20,000 Oe. To improve measurement accuracy, the resolution of the data acquisition in the range of small magnetic fields was increased.

#### 3.6.8. HPLC Analysis

The PTX concentrations for the drug release studies were determined using an HPLC method validated in our laboratory previously [71]. The analyses were made using Phenomenex Luna C-18 HPLC column (25 cm × 4.6 mm; particle size 5 μm) (Phenomenex, Torrance, CA, USA), combined with Phenomenex C-18 (4 mm × 3 mm) pre-column (Phenomenex, Torrance, CA, USA). The analyses were performed using Dionex apparatus comprising HPLC pump model 7580, DAD detector UVD 340S (Dionex, Sunnyvale, CA, USA) and Jetstream II Plus (WO Industrial Electronics, Vienna, Austria) thermostat. The other used HPLC system was combined of Varian ProStar 210 pump, Varian ProStar 410 autosampler, Varian ProStar 325 UV-Vis detector (Varian, Palo Alto, CA, USA) and Shimadzu CTO-10ASvp (Shimadzu, Kyoto, Japan) thermostat. The analyses were made in isocratic conditions; the mobile phase was a mixture of acetonitrile, methanol and water (60:2:38 (*v*/*v*/*v*) with 0.1% of trifluoroacetic acid. The flow was set as 1.00 mL/min, the column temperature was 30 °C and the injection volume was 20 μL. The chromatograms were recorded at λ = 229 nm.

#### 3.6.9. Mathematical Models for Drug Release Profiles Determination

The PTX release kinetics and the mechanism from the resulting hydrogels were both studied using zero-order, first-order, Higuchi, and Korsmeyer–Peppas models [16,72,73]. The collected drug release data were fitted to theoretical models, according to following equations:

(12) Zero order: F=kt

(13) $$\mathrm{First}\;\mathrm{order}:\;\log F=\log F_{0}-\frac{kt}{2.303}$$

(14) $$\mathrm{Higuchi}\;\mathrm{model}:\;F=k\sqrt{t}$$

(15) $$\mathrm{Korsmeyer}–\mathrm{Peppas}\;\mathrm{model}:\;F=kt^{n}(F<0.6)$$

where *F* is the fraction of drug released from the matrix after time *t*, *F*_0_ is the initial amount of drug, *k* is a model constant and *n* is the drug release exponent in the Korsmeyer–Peppas model.

#### 3.6.10. SAR Evaluation and Magnetically Induced Hyperthermia

The heat generation capability of MION in the presence of AMF was tested using an induction heating system (Dacpol, Piaseczno, Poland). The system included a 2.4 kW high frequency AC generator for induction heating (Ambrell, Rochester, NY, USA), a current, power, and sample heating time regulator, an induction coil, a water cooling system (TCW12NBSBCP0000, Texa, Pegognaga, Italy), and a power supply for the components. The MION suspension sample was placed in the center of a coil with a temperature probe (optic fiber); measurements were performed using: 360 kHz, *f* and various *H*, namely 8.2, 12.2, 20.3, 27.6, 33.8, and 36.4 kA/m. The temperature rise from 36.6 °C to 45.0 °C was measured and plotted against the time increment; cooling curves were also recorded. The collected data were used for SAR and ILP calculations, according to the slope method described in detail by Wildeboer et al. [60], and suitable for the measurements in non-adiabatic conditions. MION suspensions with predetermined nanoparticle concentrations were sonicated for 15 min prior to analysis.

## 4. Conclusions

Dual-stimuli-responsive, smart hydrogel LDDSs with desirable properties were successfully synthesized and thoroughly characterized. The PCLA copolymers were produced via ROP in the first step of this study, using Zr(acac)_4_ and PEG as a catalyst and initiator, respectively. NMR techniques were used to confirm the structure of the resulting products. The catalyst used was active in the ROP of CL and LA, leading in copolymeric products with a high monomer conversion and good agreement of the copolymer structure with theoretical assumptions. Importantly, the copolymers were synthesized with a high repeatability and narrow *Đ*, implying proper reaction control.

The microstructure of the hydrophobic PCLA blocks in the copolymer chain was found to be random, which is beneficial for biomedical applications; the PCL crystallinity was reduced, resulting in facilitated water solubility and increased hydrogel stability as compared to poly(CL)-*b*-PEG-*b*-P(CL) (PCL-PEG-PCL, PCEC) copolymer [74]. The optimal conditions for copolymerization were 130 °C for 24 h and a monomer/catalyst ratio of 750:1 mol/mol. After analyzing the water solubility and gel-forming properties of the obtained copolymers, the optimal CL/PEG and LA/PEG ratios were determined to be 1.8 and 0.3 [m/m] or 1.1 and 0.2 [m/m], respectively. The copolymers synthesized under the conditions outlined above dissolve in water and form temperature-sensitive smart hydrogels with a LCST slightly below 37 °C, making them suitable for injectable material. Furthermore, neither cyto- nor genotoxicity was observed for the synthesized matrices.

MIONs were successfully synthesized in the following step of this research using the coprecipitation method. The nanoparticles were produced form a black powder that was attracted to the strong permanent magnet. The mean diameter of the nanoparticles measured by TEM was approximately 10 nm, with a narrow size distribution, confirming a high reaction control. It was discovered that the coating agent concentration needed to be appropriately high to ensure proper coating of the MIONs and stability in aqueous media: 30% PEG 6000 was designated as optimal.

The magnetic properties of the obtained nanoparticles were assessed using a VSM. The *B*_R_ and *H*_c_ values were extremely low, and the hysteresis loops were narrow and S shaped, indicating their superparamagnetic properties. Furthermore, the M_20,000 Oe_ of the samples was relatively high and comparable to similar nanoparticles described previously in the literature.

The heating abilities of the obtained MIONs in AMF-applicable parameters (360 kHz, 12.2 kA/m) were satisfactory. The appropriate temperature increase for hyperthermia treatment was achieved quickly (<5 min) at a relatively low particle concentration of 5 mg/mL, making the particles potentially useful as a hyperthermia-inducing device.

Importantly, the PTX release profile from the PCLA-A2/MIONs/PTX smart hydrogel was prolonged and well controlled, with drug release close to zero-order kinetics. Furthermore, we discovered that the simulated hyperthermia treatment conditions had no effect on the release profiles, indicating that the developed dual-stimuli-sensitive smart hydrogel is very promising as a potential antitumor drug LDDS.

## Figures and Tables

**Table 1:** The optimization of the PCLA synthesis conditions.

Sample	Mon./Cat. Ratio ^1^ [mol/mol]	Temp. [°C]	Time [h]	*M_CL_* ^2^	*M_LA_* ^2^	*M_n_* ^2^	Conv CL [%]	Yield [%]	*M_CL_* ^3^	*M_LA_* ^3^	*M_n_* ^3^	*A_CL_* ^4^ [%]	*A_LA_*^4^ [%]	*L_Cap_* ^5^
PCLA-1	1000	110	3	1650	1650	4800	9	-	-	-	-	-	-	-
PCLA-2	1000	110	6	1650	1650	4800	20	44	400	1400	3300	27	84	1.5
PCLA-3	1000	110	24	1650	1650	4800	74	99	1800	1500	4700	106	89	2.2
PCLA-4	1000	130	3	1650	1650	4800	26	-	1500	1300	4300	91	77	5.6
PCLA-5	1000	130	6	1650	1650	4800	53	43	1200	1900	4600	74	117	1.6
PCLA-6	1000	130	6	1650	1650	4800	52	62	1200	2900	5700	75	178	1.8
PCLA-7	1000	130	24	1650	1650	4800	91	95	2100	1500	5200	129	93	2.0
PCLA-8	750	130	24	1650	1650	4800	96	82	2100	1500	5100	125	92	1.9
PCLA-9	750	130	24	1650	1650	4800	96	66	2000	1600	5000	121	94	2.2
PCLA-10	500	130	24	1650	1650	4800	98	86	2200	1500	5200	131	94	1.9
PCLA-11	250	130	24	1650	1650	4800	-	91	2100	1500	5100	129	88	1.8
PCLA-12	1000	150	6	1650	1650	4800	90	69	2800	1500	5800	168	90	1.8
PCLA-13	1000	150	24	1650	1650	4800	94	84	2100	1500	5000	125	89	2.0
PCLA-14	750	150	24	1650	1650	4800	98	56	2200	1600	5300	133	97	1.7

^1^ Monomer/catalyst molar ratio. ^2^ Number average molecular weight of CL blocks (*M_CL_*) [g/mol], LA blocks (*M_LA_*) and a copolymer chains (*M_n_*), calculated from the feed ratio. ^3^ Number average molecular weight of copolymers calculated from ^1^H NMR spectra [g/mol]. ^4^ The percent agreement between theoretical and experimental numbers of CL (*A_CL_*) and LA (*A_LA_*) mers in the copolymer chains. ^5^ Average length of the *-Cap-Cap*- sequence in the copolymer chain, calculated from ^1^H NMR spectra.

**Table 2:** The optimization of the PCLA synthesis.

Sample	CL/PEG^1^ Ratio [m/m]	LA/PEG ^1^ Ratio [m/m]	*M_CL_* ^2^	*M_LA_* ^2^	*M_n_* ^2^	*M_CL_* ^3^	*M_LA_* ^3^	*M_n_* ^3^	*A_CL_* ^4^ [%]	*A_LA_* ^4^ [%]	CL/PEG ^3^ Ratio [m/m]	LA/PEG ^3^ Ratio [m/m]	LCST ^5^ [°C]	UCST ^5^ [°C]
PCLA-15	1.8	0.3	2700	450	4650	3100	300	4900	115	71	2.1	0.2	34	41
PCLA-16	1.8	0.5	2700	750	4950	3300	600	5400	122	79	2.2	0.4	30	39
PCLA-17	2.0	0.5	3000	750	5250	3600	700	5800	119	95	2.4	0.5	30	40
PCLA-18	2.1	0.5	3150	750	5400	3600	600	5700	114	79	2.4	0.4	27	38
PCLA-19	0.9	1.2	1350	1800	4650	1700	1700	4900	128	95	1.2	1.1	29	40
PCLA-20	1.1	1.2	1650	1800	4950	2000	1600	5100	120	91	1.3	1.1	32	42
PCLA-21	1.0	1.3	1500	1950	4950	1800	1800	5100	121	90	1.2	1.2	29	39
PCLA-22	1.1	1.3	1650	1950	5100	2100	1900	5500	127	98	1.4	1.3	29	37
PCLA-23	1.2	1.3	1800	1950	5250	2000	1800	5300	109	92	1.3	1.2	29	37
PCLA-24	1.2	1.5	1800	2250	5550	2000	2200	5600	110	96	1.3	1.4	27	37
PCLA-25	1.3	1.6	1950	2400	5850	2400	2700	6600	124	113	1.6	1.8	32	40
PCLA-A1 ^6^	1.1	1.2	1650	1800	4950	2300	1600	5400	137	92	1.5	1.1	35	40
PCLA-A2 ^6^	1.8	0.3	2700	450	4650	3200	300	5000	119	70	2.1	0.2	35	41

^1^ According to feed ratio. ^2^ Number average molecular weight of CL blocks (*M_CL_*), LA blocks (*M_LA_*) and a copolymer chains (*M_n_*), calculated from feed ratio. ^3^ Number average molecular weight of polymers calculated from ^1^H NMR spectra. ^4^ The percent agreement between theoretical and experimental numbers of CL (*A_CL_*) and LA (*A_LA_*) mers in the copolymer chains. ^5^ Lower critical solution temperature (LCST) and upper critical solution temperature (UCST), determined by tube-inverting method at a polymer concentration of 25 wt.%.^6^ The samples synthesized in a larger scale.

**Table 3:** The GPC measurement results for the samples synthesized under optimal conditions.

Sample	*M_n_* [g/mol]	*M_w_* ^1^ [g/mol]	*Đ ^2^*
PCLA-15	9900	12,900	1.29
PCLA-20	9000	11,500	1.28
PCLA-A1	8400	10,600	1.25
PCLA-A2	9100	12,200	1.33

^1^ The weight average molecular weight. ^2^ Dispersity index.

**Table 4:** The synthesis of MIONs.

Sample	n Fe_2_(SO_4_)_3_ [mmol]	n FeCl_3_ [mmol]	n FeSO_4_ [mmol]	*M_n_* PEG [g/mol]	PEG Concentration [wt.%]	Fe Salts Addition Rate [mL/min]	Reaction Time [h] ^1^	Particle Size [nm] ^2^	Dispersity ^2^
S1	3.3		3.3	2000	30	0.50	1		
S2		13.2	6.6	1000	4	0.50	1	585	0.33
S3		13.2	6.6	1000	8	0.50	1	397	0.41
S4		6.6	3.3	1000	30	0.50	1	312	0.25
S5		6.6	3.3	2000	30	0.50	1	835	0.46
S6		6.6	3.3	6000	16	0.50	1	904	0.31
S7		6.6	3.3	6000	30	0.50	1	204	0.37
S8		6.6	3.3	6000	30	0.50	0	765	0.53
S9		6.6	3.3	6000	30	0.25	1	251	0.32
S10		6.6	3.3	6000	30	0.125	1	210	0.42
S11		6.6	3.3	6000	36	0.50	1	457	0.71

^1^ Reaction time after complete Fe salts addition. ^2^ Data from DLS analysis.

**Table 5:** The particle size data extracted from the TEM images.

Sample	Mean Diameter [nm]	Mean Diameter SD [nm]	Median Diameter [nm]
S2	10.3	2.2	10.1
S7	9.1	1.7	9.0
S8	9.2	1.6	9.3
S9	7.7	1.4	7.5
S10	8.8	1.3	8.6

**Table 6:** *H_c_, B_R_* and magnetization at 20,000 Oe (*M*_20,000_ *Oe*) external magnetic field recorded for the MION samples.

Sample	*H_c_* [Oe]	*B_R_* [emu/g]	*M*_20,000_*Oe* [emu/g]
S2	−10.402	1.12	67.7
S4	−2.744	0.31	65.0
S7	<0.001	<0.01	63.7
S9	−5.001	0.48	64.7

**Table 7:** SAR and ILP values of the selected MIONs.

MION Concentration [mg/mL]	*f* [kHz]	*H* [kA/m]	SAR [W/g]	ILP [nHm^2^/kg]
5	356	36.4	179.3	0.380
	356	33.8	172.5	0.423
	357	27.6	154.0	0.564
	359	20.3	119.1	0.809
	362	12.2	67.6	1.261
	368	8.2	33.5	1.356
2	356	36.4	205.3	0.435
	356	33.8	186.2	0.457
	357	27.6	154.9	0.568
	358	20.3	119.2	0.812
	362	12.2	70.8	1.320

**Table 8:** The drug release profiles from the synthesized hydrogels, analyzed using mathematical models.

	Zero-Order	First-Order	Higuchi	Korsmeyer–Peppas		
	R^2^	R^2^	R^2^	R^2^	*n*	Mechanism
PCLA-A1/PTX (phase 1)	0.8660	0.8710	0.9890	0.9880	0.517	Diffusion
PCLA-A1/PTX (phase 2)	0.9920	0.9600	0.9840	0.9890	2.417	Super Case II transport
PCLA-A2/PTX	0.9944	0.9847	0.9013	0.9761	0.690	Anomalous

**Table 9:** The fitting of the mathematical models to drug release profiles from hydrogels in simulated hyperthermia conditions.

	Zero-Order	First-Order	Higuchi	Korsmeyer–Peppas		
	R^2^	R^2^	R^2^	R^2^	*n*	Mechanism
42 °C	0.9805	0.9850	0.9753	0.9869	0.524	Diffusion
37 °C	0.9861	0.9942	0.9599	0.9908	0.796	Anomalous
37/42 °C	0.9924	0.9833	0.9559	0.9895	0.719	Anomalous

## Data Availability

The data presented in this study are available on request from the first author.

## Supplementary Materials

The following supporting information can be downloaded at: https://www.mdpi.com/article/10.3390/ijms24086906/s1.
