# Supplementary material for: Dual-Stimuli-Sensitive Smart Hydrogels Containing Magnetic Nanoparticles as Antitumor Local Drug Delivery Systems—Synthesis and Characterization
Source: Int J Mol Sci. 2023 Apr 7;24(8):6906. doi: 10.3390/ijms24086906 (PMC10138940; doi:10.3390/ijms24086906)
Supplement: Supplementary file 1 [file ijms-24-06906-s001.zip › ijms-2310993-supplementary.pdf]

International Journal of *Molecular Sciences*

MDPI

Supplementary Materials

Dual-Stimuli-Sensitive Smart Hydrogels Containing Magnetic Nanoparticles as Antitumor Local Drug Delivery Systems—Synthesis and Characterization

Adam Kasiński [1], Agata Świerczek [1], Monika Zielińska-Pisklak [1], Sebastian Kowalczyk [2], Andrzej Plichta [2], Anna Zgadzaj [3], Ewa Oledzka [1] and Marcin Sobczak [1,4,*]

[1] Department of Pharmaceutical Chemistry and Biomaterials, Faculty of Pharmacy, Medical University of Warsaw, Banacha 1 Str., 02-097 Warsaw, Poland
[2] Faculty of Chemistry, Warsaw University of Technology, 3 Noakowskiego Str., 00-664 Warsaw, Poland
[3] Department of Toxicology and Food Science, Faculty of Pharmacy, Medical University of Warsaw, 1 Banacha Str., 02-097 Warsaw, Poland
[4] Military Institute of Hygiene and Epidemiology, 4 Kozielska Str., 01-163 Warsaw, Poland
* Correspondence: marcin.sobczak@wp.pl or marcin.sobczak@wum.edu.pl

Table S1. The results of NRU cytotoxicity tests of PCLA matrices at the highest tested concentration (1 mg/mL).

Sample	Cells viability ± SD [%]
**PCLA-A1**	97 ± 2
**PCLA-A2**	107 ± 4
**PC**	2 ± 3
**NC**	108 ± 4

PC – positive control, latex 10 mg/mL.
NC – negative control, polyethylene foil 50 mg/mL.

Table S2. The genotoxicity assay results for PCLA matrices at the highest tested concentration (1 mg/mL).

Sample	-S9[1]		+S9[2]	
	G ± SD	IR ± SD	G ± SD	IR ± SD
**PCLA-A1**	1.18 ± 0.18	0.93 ± 0.10	1.24 ± 0.19	0.70 ± 0.14
**PCLA-A2**	1.12 ± 0.05	0.70 ± 0.22	1.21 ± 0.01	0.72 ± 0.02
**Solvent control**	1.02 ± 0.03	0.92 ± 0.17	1.00 ± 0.05	0.89 ± 0.09
**Positive control**	0.74 ± 0.14	12.15 ± 1.31	0.77 ± 0.07	8.41 ± 0.95
**Negative control**	1.00 ± 0.06	1.00 ± 0.23	1.00 ± 0.06	1.00 ± 0.09

[1] version without metabolic activation
[2] version with metabolic activation

Table S3. The visual observation of MIONs sedimentation in water.

Sample	Time					
	**0 h**	**2 h**	**24 h**	**48 h**	**72 h**	**168 h**
**S2**	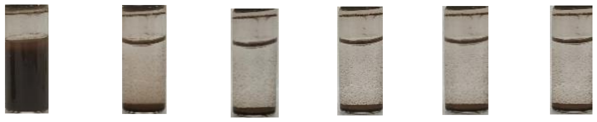					
**S4**	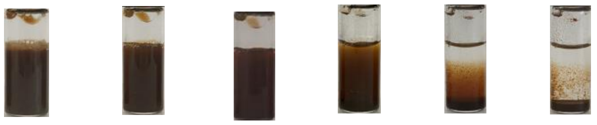					
**S5**	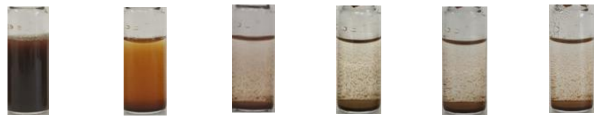					
**S6**	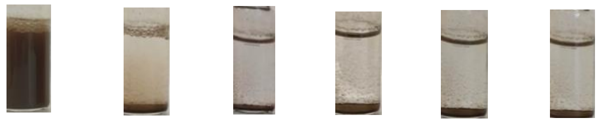					
**S7**	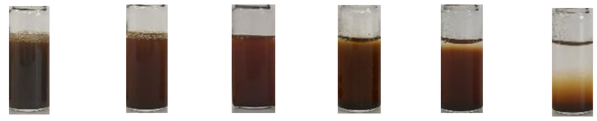					
**S8**	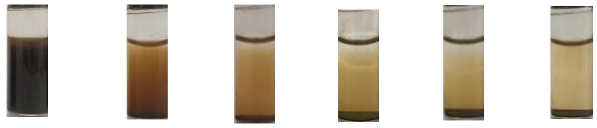					
**S9**	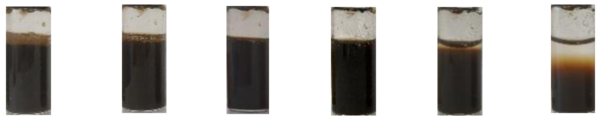					
**S10**	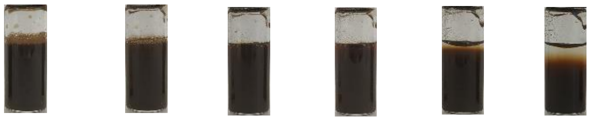					
**S11**	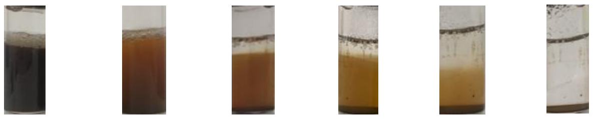					

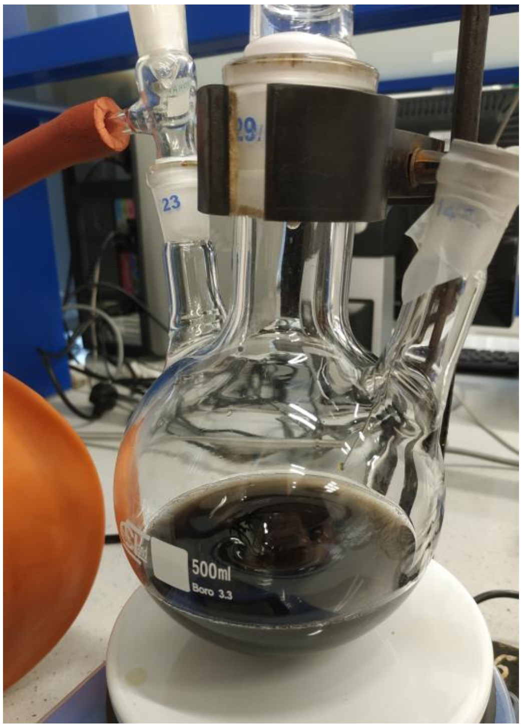

Figure S1. An illustration photo of the synthesized MIONs.

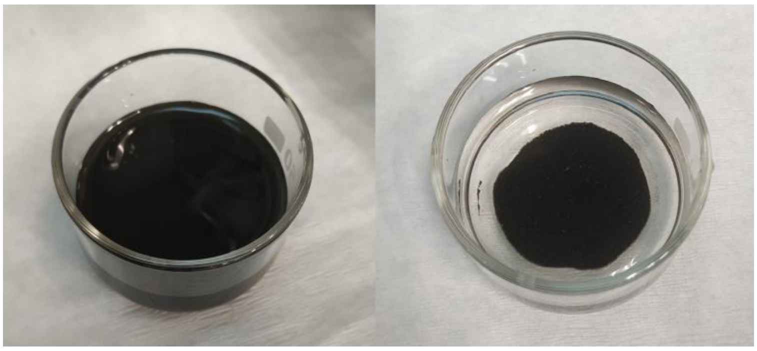

Figure S2. MIONs magnetically-assisted sedimentation.
